# Heterosis of growth trait regulated by DNA methylation and miRNA in allotriploid fish

**DOI:** 10.1186/s13072-022-00455-6

**Published:** 2022-05-21

**Authors:** Li Ren, Hong Zhang, Mengxue Luo, Xin Gao, Jialin Cui, Xueyin Zhang, Shaojun Liu

**Affiliations:** 1 State Key Laboratory of Developmental Biology of Freshwater Fish, College of Life Sciences, Hunan Normal University, Changsha, Hunan 410081 People’s Republic of China; 2 Guangdong Laboratory for Lingnan Modern Agriculture, South China Agricultural University, Guangzhou, 510642 Guangdong People’s Republic of China

**Keywords:** DNA methylation, microRNA, Allotriploid, Growth

## Abstract

**Background:**

Heterosis of growth traits in allotriploid fish has benefited the production of aquaculture for many years, yet its genetic and molecular basis has remained obscure. Now, an allotriploid complex, including two triploids and their diploid inbred parents, has provided an excellent model for investigating the potential regulatory mechanisms of heterosis.

**Results:**

Here, we performed a series of analyses on DNA methylation modification and miRNA expression in combination with gene expression in the allotriploid complex. We first established a model of *cis*- and *trans*-regulation related to DNA methylation and miRNA in allotriploids. Then, comparative analyses showed that DNA methylation contributed to the emergence of a dosage compensation effect, which reduced gene expression levels in the triploid to the diploid state. We detected 31 genes regulated by DNA methylation in the subgenomes of the allotriploids. Finally, the patterns of coevolution between small RNAs and their homoeologous targets were classified and used to predict the regulation of miRNA expression in the allotriploids.

**Conclusions:**

Our results uncovered the regulatory network between DNA methylation and miRNAs in allotriploids, which not only helps us understand the regulatory mechanisms of heterosis of growth traits but also benefits the study and application of epigenetics in aquaculture.

**Supplementary Information:**

The online version contains supplementary material available at 10.1186/s13072-022-00455-6.

## Background

Allotriploids, including two or more different genomes in somatic cells, always exhibit phenotypic differences in growth [1, 2], gonadogenesis [3], and innate immunity [4, 5] from diploid relatives or parents. Some phenotypic diversities, such as heterosis, provide us with a useful research resource for investigating their potential mechanisms and could improve agricultural production by engineering breeding [6, 7]. Some potential molecular regulatory mechanisms involving genomic [8] and transcriptomic changes [9, 10] have been reported as key factors in shaping heterosis. However, studies on epigenetic modification and post-transcriptional modification are very rare in allotriploids. How modifications of homoeologs affect phenotypes in allotriploids is interesting but largely unknown.

*Carassius auratus* red var. and *Cyprinus carpio* L., belonging to different genera of the subfamily Cyprinidae, experienced a common whole-genome duplication event approximately 13.75 million years ago (Mya) [11]. Their divergence time was dated to 10.0 Mya [11]. As important aquaculture species in China, the annual production capacity of common carp and crucian carp in 2018 was approximately 2.9 and 2.7 million tons, respectively [12]. Common carp and crucian carp are mainly distributed in Asia and Europe, reaching as far as the Arctic Circle. Their distinct morphological characteristics, including body color and shape [13, 14], contribute to their extensive adaptability to a diversified environment of slow-moving rivers, lakes, and ponds. Heterosis of the growth trait was observed in the allotriploid complex of common carp and crucian carp [7, 9]. Among the two triploids, 3nRC_2_ had two sets of subgenome C (originating from common carp) and one set of subgenome R (originating from red crucian carp) [7, 9], while 3nR_2_C had one set of subgenome C and two sets of subgenome R [7, 9].

Dosage compensation is always detected in the two X chromosomes of female humans, leading to the silencing of one of the X chromosomes [15]. Moreover, some studies have reported dosage compensation in allotriploid animals and plants, reducing transcript levels to the diploid state [1, 16, 17]. Incomplete dosage compensation and maternal effects were predicted in the two triploid fish (3nR_2_C and 3nRC_2_) based on the gene expression changes between the inbred parents and the two triploids [9]. This also had various effects on the expression of growth-regulated genes, resulting in heterosis of the growth trait. However, the effects of epigenetic regulation on these expression changes are still unknown. Dosage compensation on the sex chromosomes is not only regulated by DNA methylation [18] but is also affected by microRNAs (miRNAs), noncoding RNAs that regulate gene expression at the post-transcriptional level [19, 20]. However, the expansion of miRNA families and their target sequences, arising through gene duplication, complicates the miRNA–mRNA regulatory network in allotriploids. Although some studies have reported miRNA expression changes between triploid and diploid individuals, the expression of miRNAs and the target genes did not distinguish between different homoeologs in subgenomes [21]. The two homoeologs in 3nR_2_C and 3nRC_2_ originate from species of different genera and exhibit nucleotide differences in parts of the coding sequences and their regulatory regions. Therefore, the specific recognition and detection of mRNA and miRNA expression levels and DNA methylation loci will improve our understanding of the regulatory interactions between the two subgenomes in intergeneric hybrids.

The specific genetic status of the two allotriploids (3nR_2_C and 3nRC_2_) offers us a new insight into how dosage compensation occurs between two sets of the same chromosomes [9, 16, 17], and helps us further understand the relationship between genetic changes and heterosis of growth traits in allotriploids. Here, we performed DNA methylation sequencing (methyl-seq), miRNA-seq, and mRNA-seq in the muscles of the two triploids and their inbred parents (*C. auratus* red var. and *C. carpio* L.). Integrating analyses of gene expression with DNA methylation and miRNA expression, the changes between diploids and triploids or between subgenomes of 3nR_2_C and 3nRC_2_ will help us investigate potential mechanisms of growth traits in the triploids and improve the aquaculture of common carp and crucian carp by epigenetic engineering and breeding.

## Results

### Heterosis of growth traits in allotriploid fish

**Fig. 1:**
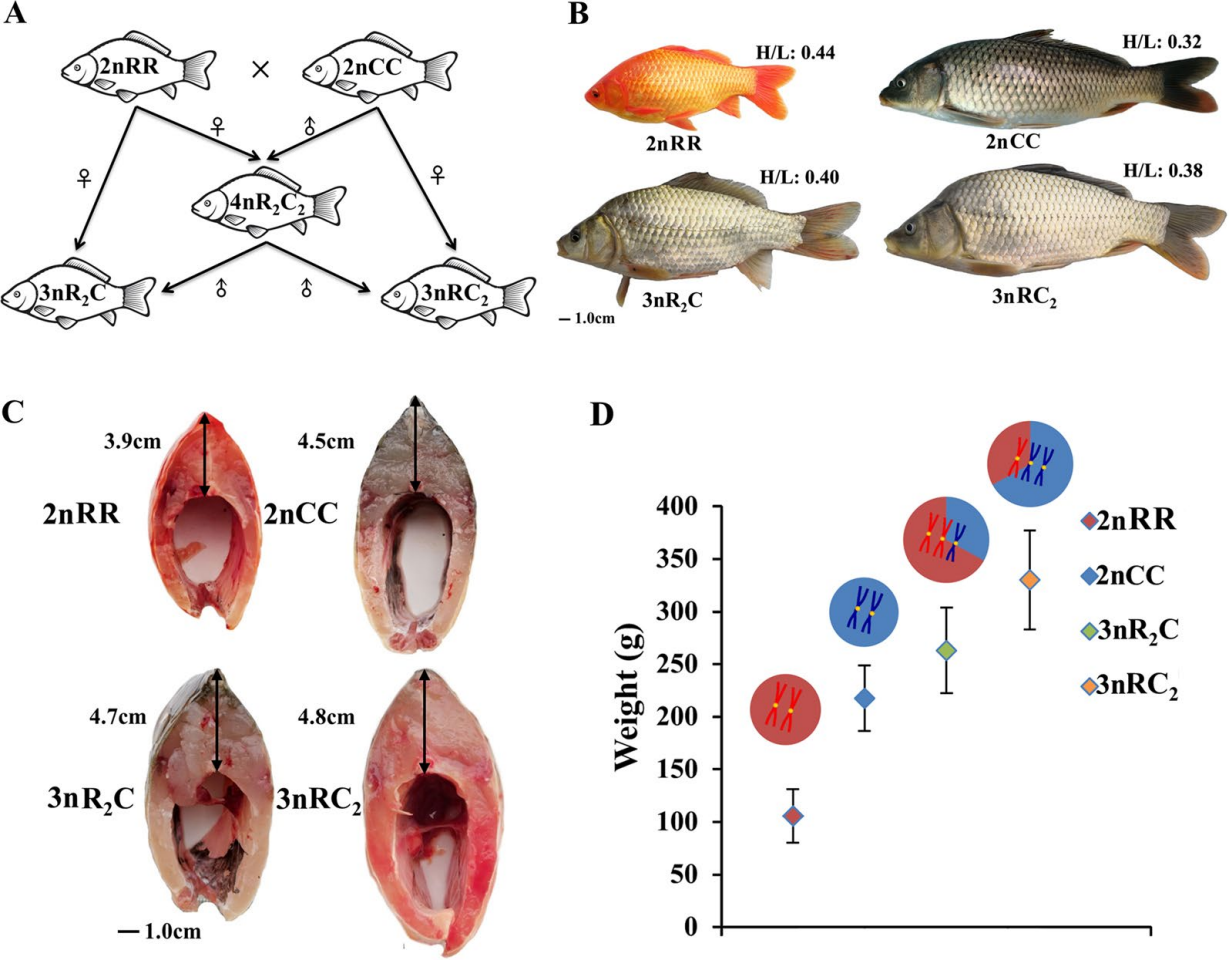
Heterosis of growth traits in the two triploid fish (8 months after hatching). **A** The procedure for the two allotriploids (3nR_2_C and 3nRC_2_). **B** The appearance of the two triploids and their inbred parents. The ratio of average body height and body length (H/L) is marked. **C** The height of the back muscle. **D** The genotypes and weights of the four fish

In our studies, the two triploids (3nR_2_C and 3nRC_2_) were obtained by backcrossing female allotetraploids of *C. auratus* red var. and *C. carpio* L. (4nR_2_C_2_) with male *C. auratus* red var. (2nRR) and *C. carpio* L. (2nCC), respectively (Fig. 1A) [7, 9]. Eight months after hatching, some growth traits, including body length (BL), body height (BH), height of back muscle (HBM), and body weight (BW), showed higher growth rates in the two triploids (3nR_2_C and 3nRC_2_) than in the diploid inbred parents (2nRR and 2nCC) (Fig. 1). Among these traits, similar values in HBM were observed between the two triploids (Fig. 1). Moreover, the ratios of BL vs. BH in the two triploids exhibited intermediate characteristics between the inbred parents (Fig. 1). Specifically, the two triploids exhibited a predilection for their maternal parents for their distinct growth phenotypes involving HBM and ratios of BL vs. BH (Fig. 1A, B). These differences may be related to their genotypes and play a key role in heterosis.

### *Cis*- and *trans-*regulation models of DNA methylation modification and miRNA regulation

**Fig. 2:**
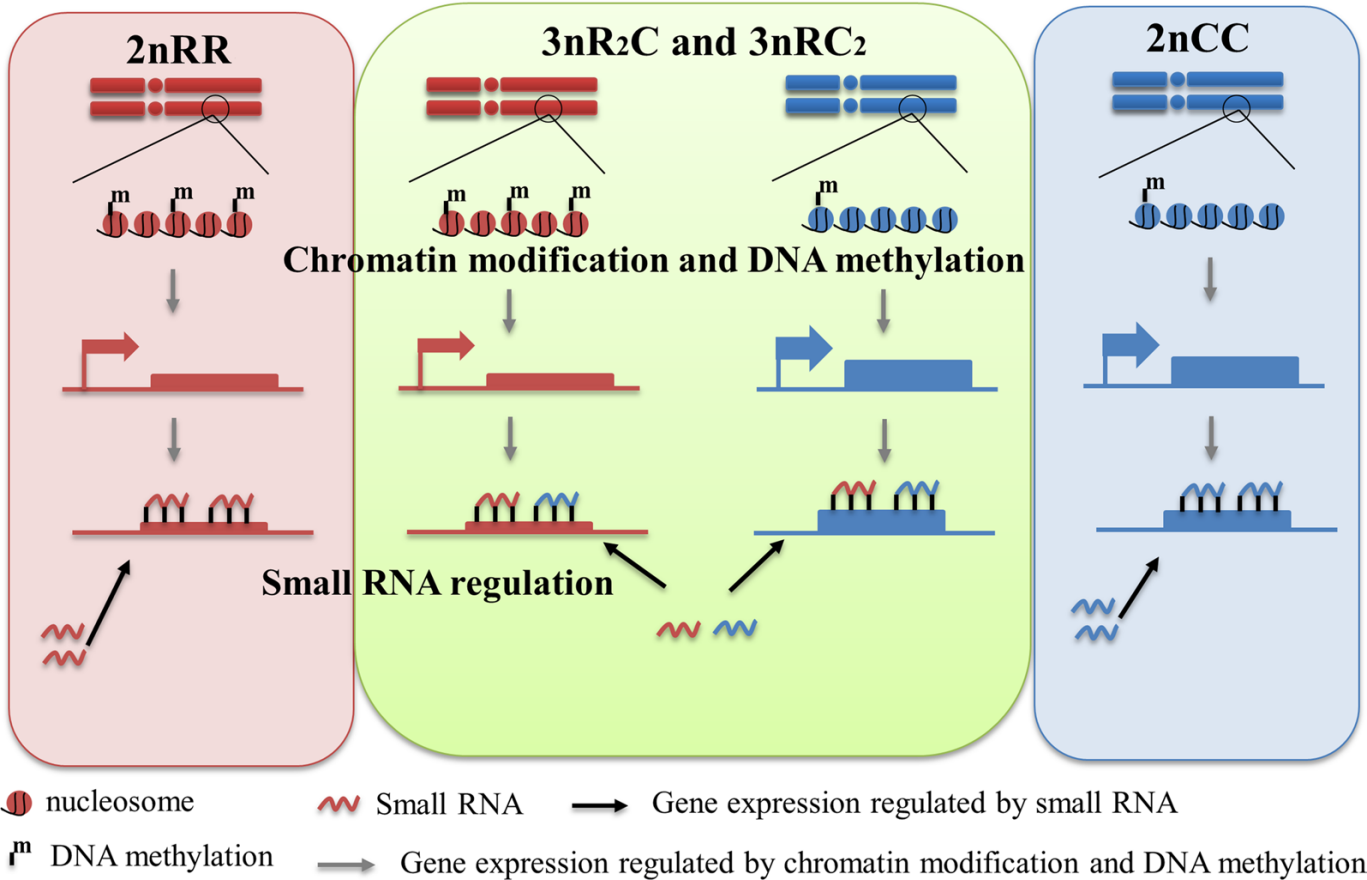
A model for gene expression mediated by DNA methylation and miRNA in the allotriploid complex of red crucian carp (2nRR) and common carp (2nCC). In the hybrid system, the small RNAs originating from subgenomes R and C regulated the expression of homoeologous genes in the triploids. A similar regulatory model was described in *Arabidopsis* allotetraploid [22, 23]

To investigate potential genetic mechanisms and their roles in the heterosis of growth trait heterosis, methyl-seq, miRNA-seq, and mRNA-seq data were obtained from 3nR_2_C, 3nRC_2_, and the two inbred parents. The complex regulation of gene expression in the two allotriploid fish led us to establish a model for investigating *cis*- and *trans-*regulation in DNA methylation modification and *trans*-regulation in miRNA regulation. In the allotriploid system, the expression of homoeologs R (originating from 2nRR) and C (originating from 2nCC) was not just regulated by their respective methylation modifications but also by similar miRNA systems in the two subgenomes (Fig. 2). The development of diagrammatic regulatory systems aided us in better understanding *cis*- and *trans*-regulation in allotriploids, as well as their effects on phenotype. *Cis*- and *trans-*regulation in hybrids was assessed as described by Wang et al. [22, 23]. The changes in DNA methylation levels and the expression of their regulated genes from the inbred parents to their triploids are used to assess DNA methylation-mediated *cis*-regulation. MicroRNA-mediated *trans*-regulation in hybrids is then regulated by *trans*-acting factors involving microRNA, which was assessed by comparing the change trends in microRNA expression and targeted gene expression between inbred parents and their triploids. The combined genomes of 2nRR and 2nCC were used as a reference genome for the two triploid fish in our subsequent analyses for three reasons: (1) the lack of an assembly genome for the triploids; (2) completed sets of original chromosomes from the two inbred parents [9]; and (3) research on conserved homoeologous genes in subgenomes. Therefore, analyses of orthologous gene pairs between 2nRR and 2nCC, and homoeologous gene pairs between subgenomes R and C in the two triploids (3nR_2_C and 3nRC_2_), were performed to study the interactions between DNA methylation and miRNA and mRNA expression in the triploids.

### Methylation divergence between subgenomes R and C

To uncover DNA methylation differences from diploids to allotriploids, we performed whole-genome bisulfite sequencing (WGBS) in the muscles of 2nRR, 2nCC, 3nR_2_C, and 3nRC_2_ fish. After mapping the reads of the DNA methylation data to the 2nRR and 2nCC combined genome, 270.85 Gb (48.91%) of data uniquely mapped in the two triploids (Additional file 1: Table S1). Based on the mapped results, 138.39 Gb uniquely mapped data (49.33% of all methylation data) were detected in the three 2nCC samples, while 150.85 Gb data (54.73%) were found in 2nRR (Additional file 1: Table S1). Focusing on cytosines, the methylation levels in CpG islands (70.72%) were higher than those in CHH (0.30%) and CHG (0.30%) across all samples. However, the levels of methylation divergence at CpG and nonCpG sites were conserved among the two triploids and the inbred parents (Additional file 1: Table S2).

Although similar genome sizes were detected between the 2nRR (1.42 Gb) and 2nCC (1.49 Gb) genomes, different distributions of transposable elements (TEs) between them reflected that TEs had contributed to the diversified species divergence after a common allotetraploidization in their ancestors [11, 24] (Additional file 1: Table S3 and Fig. S1A). More TEs were found in 2nRR (38.99%) than in 2nCC (34.76%). The top three TE difference ratios between 2nRR and 2nCC were observed in orthologous chromosome pair (OCP) 43 (16.75%), 2 (10.04%), and 5 (10.06%). Meanwhile, high DNA methylation was observed in the region of the TEs (Additional file 1: Fig. S1B and C). We first identified global TE methylation levels and found that the TE methylation levels in 3nR_2_C trended toward 2nRR, while those in 3nRC_2_ trended toward 2nCC (Additional file 1: Fig. S1B). Then, we further detected the TE methylation levels in different subgenomes in the triploids and the respective ones in the inbred parents. Higher TE DNA methylation was observed in both subgenomes of 3nR_2_C than in 3nRC_2_ (Additional file 1: Fig. S1B).

**Fig. 3:**
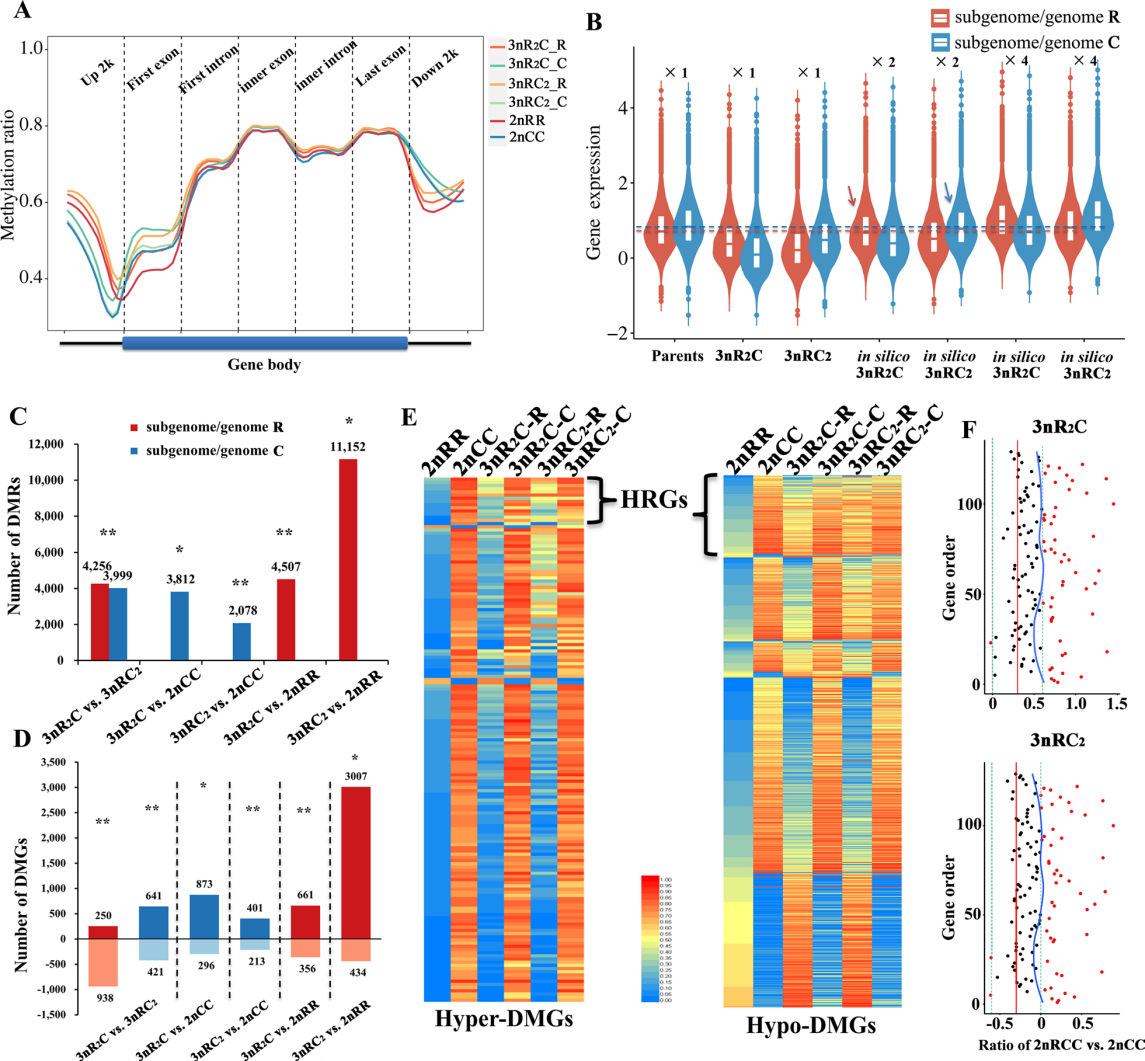
Incomplete dosage compensation regulated by DNA methylation levels. **A** DNA methylation levels of different gene elements in the subgenome of the two triploids and the inbred parents. Each region was divided into twenty bins based on total lengths. **B** Assessments of dosage compensation based on the different in silico values of homoeolog expression of the two triploids compared with those of their inbred parents. The actual values of two homoeolog expression in the triploids are marked as “ × 1.” Duplicated values of two homoeolog expression are marked as “ × 2.” Fourfold values of two homoeolog expression are marked as “ × 4.” **C** Differentially methylated regions (DMRs) in the two triploids and their inbred parents. **D** The distribution of differentially methylated genes (DMGs), for which the DMRs were located in the region of “Up in 2 k.” Crimson and dark blue represent higher methylation levels in the former comparison, while light red and light blue represent higher methylation levels in the latter comparison. The symbol “*” represents unequal numbers of gene copies between subgenomes R and C, while the symbol “**” represents equal numbers. **E** The potential homoeologous recombinant genes (HRGs), led by the exchange of subgenomes R and C, were predicted by the decrease of the difference in the methylation ratio between two homoeologs in both the triploids in comparison to the inbred parents. **F** Recombination events between the 2nRR and 2nCC subgenomes were observed in HRGs of 3nR_2_C and 3nRC_2_ based on whole-genome resequencing data. Red solid line represents in silico values of the ratio of 2nRR vs. 2nCC (log_10_(2) = 0.30103 in 3nR_2_C, log_10_(0.5) =  − 0.30103 in 3nRC_2_), which were calculated based on the gene copy number of 2nRR and 2nCC in each homoeologous gene pair of the two triploids. Green dashed line represents the values of the significant ratio of 2nRR vs. 2nCC (log_10_(1) = 0 and log_10_(4) = 0.60206 in 3nR_2_C; log_10_(0.25) =  − 0.60206 and log_10_(1) = 0 in 3nRC_2_), which were calculated based on the gene copy number of 2nRR and 2nCC in each homoeologous gene pair of the two triploids. Dots represent the values of the ratio of 2nRR vs. 2nCC (log_10_(x)), which were obtained from the read number (the average number in a 1 kb region) of 2nRR and 2nCC in each homoeologous gene pair of the two triploids. The blue solid line is obtained from the treatment of values (black dots) with method “loess” in ggplot2. Black dots are distributed between the two green lines, while the red dots (51 in 3nR_2_C and 50 in 3nRC_2_) are allocated outside of the two green lines

Genome-wide analyses showed that methylation levels were higher in 2nRR than in 2nCC (*p* < 0.01 in *t*-test, Fig. 3A and Additional file 1: Table S4). These results showed that DNA methylation divergence occurred between 2nRR and 2nCC after their shared ancestral allotetraploidization [11, 24]. Meanwhile, these DNA methylation divergences in the inbred parents were also observed between subgenomes R and C in the two triploids (Fig. 3A and Additional file 1: Table S4). These results suggest high heritability for DNA methylation. Then, the DNA methylation differences between the four fish were mainly enriched in the first exon coding region, 2 kb upstream of the transcription start site (TSS), and 2 kb downstream of the transcription termination site (TTS) (Additional file 1: Fig. S2). Moreover, clustering analysis in the subgenome showed that subgenome R in the two triploids clustered with 2nRR, yet subgenome C clustered with 2nCC (Additional file 1: Fig. S2). Similar to the TE methylation levels, the methylation levels of 2 kb upstream of TSS (Up 2 k) and 2 kb downstream of TTS (Down 2 k) were higher in the two triploids than in the inbred parents (*p* < 0.01), except those in subgenome C of 3nRC_2_, in which the DNA methylation levels were close to those of the parental 2nCC (Fig. 3A). These results shed light on high DNA methylation and low transcriptional efficiency in triploids.

### Incomplete dosage compensation led by DNA methylation

Recent studies have reported incomplete dosage compensation [9]. To investigate whether DNA methylation is a potential cause of this, we performed integrated analyses of differential methylation and gene expression. First, genotype analysis showed two sets of 2nRR chromosomes and one set of 2nCC chromosomes in 3nR_2_C (genotype type: AAB), and two sets of 2nCC chromosomes and one set of 2nRR chromosomes in 3nRC_2_ (genotype type: ABB) [9]. The two sets of R genomes/subgenomes were in 2nRR and 3nR_2_C, whereas the two sets of C genomes/subgenomes were in 2nCC and 3nRC_2_. However, the gene expression of R homoeologs in 3nR_2_C (× 1 in 3nR_2_C) was significantly lower than that in 2nRR (× 1) (*p* < 0.01 in *t*-test, Fig. 3B). Based on the same regulatory systems, we doubled the gene expression values of the R and C subgenomes in the two allotriploids (3nR_2_C and 3nRC_2_) (× 2). Then, we found that the doubled values of the R subgenome in 3nR_2_C (× 2 in 3nR_2_C) were equal to those in 2nRR (× 1) (*p* > 0.05, Fig. 3B). A similar phenomenon was observed between the doubled values of the C subgenome in 3nR_2_C (× 2 in 3nRC_2_) and those in 2nCC (× 1) (*p* > 0.05, Fig. 3B). So, we inferred that the four sets of R and C subgenomes in 3nR_2_C and 3nRC_2_ can transcribe the same dosage of mRNAs as the parental 2nRR and 2nCC based on the regulatory models in the two triploids.

In analyses of differentially methylated regions (DMRs) between the two triploids and their inbred parents, most DMRs in subgenome R were detected in the comparison “3nRC_2_ vs. 2nRR” (11,152), while the fewest DMRs were observed in the comparison “3nR_2_C vs. 2nRR” (Fig. 3C). In subgenome C, most DMRs were observed in the comparison “3nR_2_C vs. 3nRC_2_” (3,999), while the fewest DMRs were observed in the comparison “3nRC_2_ vs. CC” (Fig. 3C). Obviously, many DMRs were distributed between the unequal subgenomes of R and C. In the comparison “3nR_2_C vs. 3nRC_2,_” we observed that more gene copies always led to higher methylation levels in the triploids (Fig. 3C, D). Specifically, these effects were more obvious in subgenome R than in subgenome C (Fig. 3C). In the comparisons of triploids and diploids, higher methylation levels and a greater number of DMGs reflected that whole genome-wide methylation modification inhibited transcription of both homoeologs R and C and led to the triploid expression levels being close to the diploid states.

### DNA methylation changes accompanied by homoeologous recombination

We identified 165 hypermethylated (DMGs_A–B_ > 0.6) and 740 hypomethylated genes (0.4 < DMGs_A–B_ < 0.6) based on the methylation differences between 2nRR and 2nCC. Among these genes, 15 hyper-DMGs and 114 hypo-DMGs were shared in both the triploids, suggesting that the potential DNA methylation changes may have originated from the parental 4nR_2_C_2_ (Fig. 3E). These DMGs were considered potential homoeologous recombinant genes (HRGs) because homoeologous recombination (HR) always leads to a decrease in DNA methylation divergence between homoeologs R and C in promoter regions (Fig. 3E). For assessing the accuracy of HRGs predicted by DNA methylation, the mapped read number of homoeologs R and C was obtained from whole-genome resequencing, and unequal HRs were detected in some genes (52 in 3nR_2_C, 50 in 3nRC_2_, red dots, Fig. 3F) based on the ratios of the homoeologs R vs. C [25]. Meanwhile, a similar shape (blue line, Fig. 3F) between 3nR_2_C and 3nRC_2_ indicated a similar distribution of HRs in these genes, suggesting that the prediction that the HRs were inherited from paternal 4nR_2_C_2_ was highly accurate.

### Gene expression regulated by DNA methylation

**Fig. 4:**
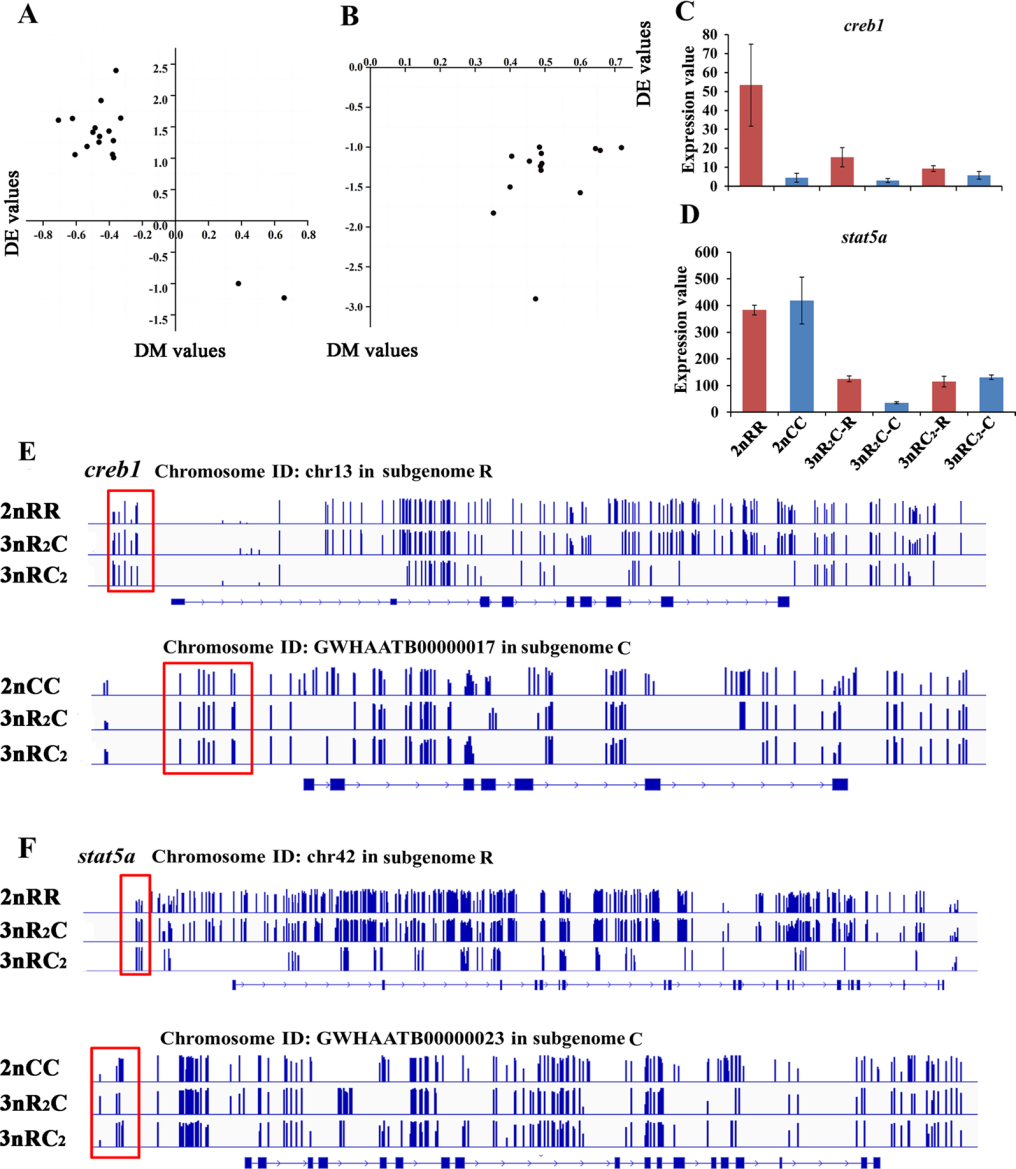
Gene expression regulated by DNA methylation. **A** Negative correlation between values of differential expression (DE) and differential methylation (DM) in subgenome R of the two triploids and 2nRR. **B** Negative correlation between DE and DM values in subgenome C of the two triploids and 2nCC. **C** The expression of *CREB1*. **D** The expression of *STAT5A*. **E** The methylation level of *CREB1* was higher in homoeologs of the two triploids than in their inbred parents. **F** The methylation level of *STAT5A* was higher in homoeologs of the two triploids than in their inbred parents

DNA methylation in the promoter region is a major epigenetic modification and is always negatively correlated with gene expression. In the hybrid system of the two triploids, 17 and 14 genes showed negative correlation between differential expression (DE) and differential methylation (DM) in subgenomes R and C, respectively (Fig. 4A–B). These genes contributed to multiple functions, including regulation of response to stimulus and cellular metabolic process (Additional file 1: Fig. S3). Moreover, comparing the two triploids with their inbred diploid parents, various genes were identified with negative correlations between the DE and DM values (red dots, Additional file 1: Fig. S4), while other genes showed a positive correlation (black dots, Additional file 1: Fig. S4). Integrated analyses of methylation and gene expression changes demonstrated that only a few genes were regulated by DNA methylation, while most of the expressed genes might be regulated by *trans-*regulation in the hybrid systems.

### Patterns of miRNA regulation

**Fig. 5:**
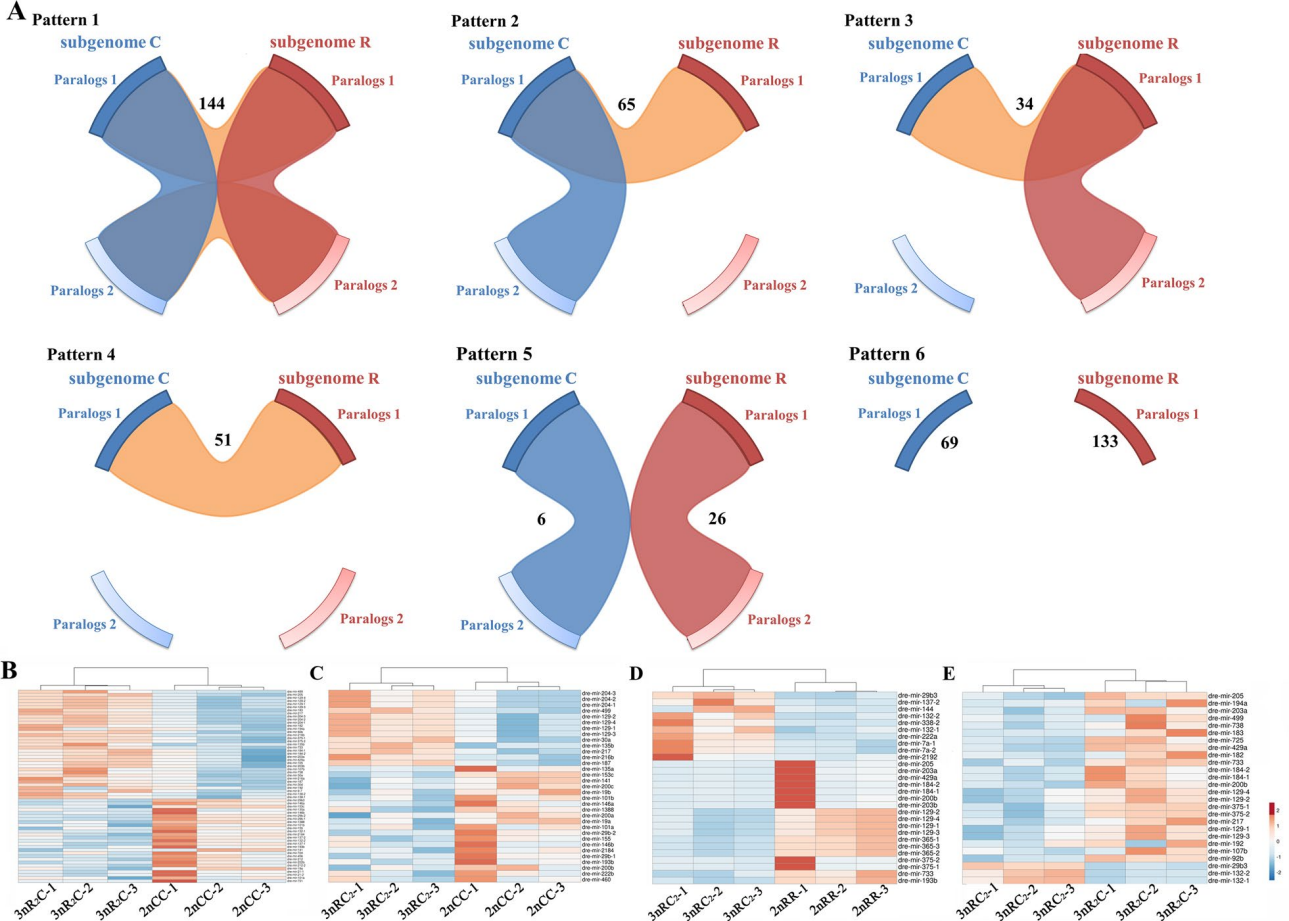
miRNA expression in allotriploids. **A** Schematic diagrams of miRNA patterns in the triploids. “Pattern 1” represents the same miRNA allocated to two locations in both subgenomes R and C. “Pattern 2” represents the same miRNA detected in one location in subgenome R and two locations in subgenome C. “Pattern 3” represents the same miRNA detected in two locations in subgenome R and one location in subgenome C. “Pattern 4” represents the same miRNA allocated to one location in both subgenomes R and C. “Pattern 5” represents the same miRNA allocated to two locations in subgenome R or C. “Pattern 6” represents the miRNA specifically detected in one location in subgenome R or C. The number of miRNAs in each pattern is marked in the figure. **B–E** DE of miRNAs among the four fish

In a hybrid system, miRNA regulation is a key trans-regulatory mechanism that may play a dominant role in gene expression regulation. We obtained 129.09 million clean reads (2.91 Gb) from small RNA-seq from the four fish with three biological replicates (Additional file 1: Table S5). Among these data, 121.10 million reads mapped to the combined genomes of the inbred parents, and 258 miRNAs were detected in 2nRR, 2nCC, and the two triploids using the zebrafish miRNA. The multiple transcription sites of some miRNAs were not just detected in 2nRR and/or 2nCC but also in the subgenomes of R and C in the two triploids. Under these conditions, we established six patterns for classifying these conserved miRNAs, which might result from random duplication of chromosome regions or whole-genome duplication (Fig. 5A). For the allotriploids, miRNAs in Pattern 1 (114) and Pattern 4 (51) possessed four and two copies, respectively, that were evenly distributed in subgenomes R and C (Fig. 5A). In addition, three copies were observed in 65 (Pattern 2) and 34 (Pattern 3) miRNAs, in which two miRNA copies were distributed in subgenome C or R, respectively, and the other copy was distributed in the other subgenome (Fig. 5A). The miRNAs in Pattern 5 had two copies distributed in only subgenome R (26) or subgenome C (6) (Fig. 5A). Pattern 6 describes miRNAs with only a single copy in subgenome R (133) or C (69) (Fig. 5A). The evolutionarily conserved class of miRNAs sheds light on the relationship between miRNA copy number and their expression.

Because of the evolutionarily conserved miRNAs, the expression of each miRNA was calculated by combining the expression values of subgenomes R and C based on the mapped files and annotation results. The analysis of differential expression (DE) was performed on 256 miRNAs, of which 33 miRNAs were detected between 3nRC_2_ and 2nCC, and 62 miRNAs were detected between 3nR_2_C and 2nCC (Fig. 5B, C and Additional file 1: Fig. S5A). DE was detected in 28 miRNAs between 3nRC_2_ and 2nRR, and for only two miRNAs between 3nR_2_C and 2nRR (Fig. 5D and Additional file 1: Fig. S5A). Meanwhile, 26 miRNAs showed DE between 3nRC_2_ and 3nR_2_C (Fig. 5E). Among these miRNAs, dre-mir-182 and dre-mir-183 showed DE in the three comparisons (Additional file 1: Fig. S5B).

### Contribution of DNA methylation modification and miRNA regulation to heterosis of growth traits

Our aims were to investigate the genetic mechanisms of DNA methylation and miRNA in triploids and to further understand their potential effects on heterosis of growth traits. The functional distribution of the hyper-/hypo-DMGs was performed and annotated with Gene Ontology databases and 97 pathways (Additional file 1: Table S6 and Fig. S6). Among these genes, six genes were enriched for the growth hormone synthesis, secretion, and action pathway (map04935), while nine genes were distributed in the insulin secretion pathway (map04911) (Additional file 1: Figs. S7, 8). Meanwhile, high methylation levels led to the expression silencing of homoeolog C in *ATF4* and *SNAP25* of the two triploids, while low methylation levels and expression of homoeolog R were detected (Fig. 4E). On the contrary, low methylation levels of homoeolog R were observed in *CREB1* and *STAT5A*, while high methylation levels were detected in homoeolog C (Fig. 4E). Some growth-regulated genes were regulated by miRNA regulation, especially in the growth hormone synthesis, secretion, and action pathway (map04935). Among these growth-regulated genes, the expression levels of *P85B* and *EP300* were regulated by mir-205, which showed DE between the two triploids (Fig. 5E). However, no DE of the two genes between the two triploids indicated a complex regulation system in the triploids. The heterosis of growth traits in the triploids might involve cooperative changes to a series of growth-regulated genes.

## Discussion

Both common carp and crucian carp are important aquaculture species in China because of their wide distribution and adaptation [12–14]. The two triploid fish, which have been obtained via the interploid crossing of 4nR_2_C_2_ with its inbred parents (common carp and red crucian carp), are also important aquaculture varieties in the Yangtze River because of their stronger innate immunity and faster growth rate than their inbred parents (Fig. 1) [7, 9]. To investigate the potential regulatory mechanisms in the triploids and their effects on the growth traits, we focused on *cis*- and *trans*-regulation (DNA methylation modification and miRNA regulation) in combination with gene expression (Fig. 2).

The mRNAs and miRNAs had more gene copies in the triploids than in the diploid inbred parents, while heterosis of the growth rate and body size was observed in the triploids (Fig. 1). However, a dosage compensation effect caused the gene expression of the three sets of subgenomes in the triploids to be close to those of the two sets of subgenomes in the inbred parents (Fig. 3). This was regulated by DNA methylation and other regulatory mechanisms, and effectively balanced the steady state of cells and individuals [9, 17]. However, the gene expression changes caused by the effect were not identical. Higher expression of *GH* and *IGF1* in the triploid progenies compared with that in the diploid parents may contribute to the heterosis of growth [26]. Meanwhile, the coordinated regulation of DNA methylation and miRNA expression contributed to various effects on the expression of targeted growth-regulated genes (Figs. 2–5), which may be related to the heterosis of growth in the triploid fish.

Specific genotypes (two sets of subgenomes R and one set of subgenome C in 3nR_2_C, one set of subgenome R and two sets of subgenomes C in 3nRC_2_) and different growth phenotypes also shed light on the complex regulatory mechanisms of heterosis of growth traits. Functional divergences between the two inbred parents (such as growth rate and body size, 2nCC > 2nRR) revealed their divergent regulatory systems (Figs. 1–5). In the hybrid system of the triploids, the expression of targeted mRNAs was regulated by miRNAs originating from both subgenomes R and C [16, 27, 28]. Comparative analysis of the two allotriploids revealed that more gene copies of subgenome C may contribute to the expression bias of homoeolog C and further benefit the large body size and rapid growth rate (Fig. 1). Under these conditions, exchanges of the promoter region between subgenomes R and C could disturb the DNA methylation originating from the two inbred parents and further shape the diversified expression of homoeologs (Fig. 3) [25, 29]. However, the dispersed distribution of these recombination events in chromosome segments may result from DNA damage response and repair (via recombination) [29]. Additional studies on the recombination mechanisms in the allotetraploid fish are necessary. Meanwhile, various changes in miRNA expression from the inbred parents to the allotriploid progeny also contributed to the complexity of homoeolog expression (Fig. 5).

## Conclusions

In the triploids, gene expression changes from the inbred parents and expression bias of homoeologs play a key role in heterosis of growth [9]. Meanwhile, the different heterosis of growth traits may be relative to the different dosage of R and C subgenomes in the two triploids (2:1 in 3nR_2_C and 1:2 in 3nRC_2_), which derive from the distinctly different body sizes and ratios of BL vs. BH between 2nRR and 2nCC. A significant effect of different subgenomes on heterosis was also described in triploid maize [30]. These expression changes were regulated by complex *cis*- and *trans*-regulatory mechanisms, including DNA methylation and miRNAs. Our results help us to further understand the potential regulatory mechanisms of heterosis of growth traits in triploid fish.

## Methods

### Animal materials

The fish used in this study were diploid *C. auratus* red var. (2nRR), diploid *C. carpio* L. (2nCC), an triploid fish (3nR_2_C) obtained from hybridization between *C. auratus* red var. (♀) and an allotetraploid of *C. auratus* red var. x *C. carpio* L. (4nR_2_C_2_, ♂), and an triploid fish (3nRC_2_) obtained from hybridization between *C. carpio* (♀) and 4nR_2_C_2_ (♂). The fish were fed in a pool located in the Engineering Center of Polyploidy Fish Breeding of the National Education Ministry, Hunan Normal University, China. Two-year-old male individuals of each fish were collected with three biological replicates. These fish were deeply anesthetized with 300 mg/L Tricaine Methanesulfonate (Sigma-Aldrich, St Louis, MO, USA) for 10 min (25 °C) in a separate tank. After confirming death, they were collected from the water for dissection. A flow cytometer was used to measure the DNA content of the erythrocytes. To avoid contamination, all tissue samples were collected using sterilized scissors and tweezers.

### Identification of orthologous chromosomes and orthologous genes

Despite the lack of a reference genome for the nascent triploids, the combined genome of the two inbred parents could still be used as the in silico genome because of the presence of the two subgenomes of the inbred parents in the triploids. Therefore, the combined genome was used as the reference genome for investigating DNA methylation and microRNA (miRNA) and mRNA expression. Then, orthologs between *C. auratus* red var. and *C. carpio* L. were considered the homoeologs of subgenomes in the two triploids (3nR_2_C and 3nRC_2_). Orthologous chromosomes between *C. auratus* red var. and *C. carpio* L. were detected using MCScanX with the thresholds of e-value < 10^−5^ and max gaps < 25 [31]. We performed reciprocal highest scores in BLAST similarity searches of all annotated genes of *C. auratus* red var. and *C. carpio* L. with the criteria of e-value < 10^−5^, a minimum of 80% sequence length coverage, and 300 bp length of sequence identity. Then, high confidence orthologous gene pairs were finally identified based on the shared gene pairs satisfying the results of the reciprocal BLAST and orthologous chromosomes.

### Whole-genome bisulfite sequencing and mapping of methyl-seq reads

The WGBS libraries were constructed following the standard protocol [32]. Briefly, genomic DNA spiked-in with 0.5% unmethylated lambda DNA (Fermentas) was first fragmented into 200–300 bp fragments using a Covaris S220 system. After being concentrated, the sheared DNA was end-repaired, dA-tailed, and ligated with pre-methylated TruSeq DNA adapters (Illumina). Bisulfite conversion was conducted with an EZ DNA Methylation™ Kit (Zymo Research). After the bisulfite conversion, the converted templates were PCR amplified and quantified with Qubit 2.0. Then, the length of the insert fragment in the libraries was detected by Agilent 2100, and the effective concentration (> 2 nM) was quantified by qPCR assay. The final quality-ensured libraries were sequenced on a NovaSeq 6000 Sequencing System with paired end reads (2 × 150 bp) according to the standard protocol.

After quality checking of the methyl-seq reads, the clean reads of *C. auratus* red var. and *C. carpio* L. were mapped to the respective genomes, and the clean reads of the two triploids were mapped to the combined genome sequences of *C. auratus* red var. and *C. carpio* L. (Yellow River carp) [11, 24]. The Bismark analysis pipeline was used to detect the methylated loci with the mapped parameters (–score_min L, 0, − 0.2 − X 1000 –no-mixed–no-discordant) [33, 34]. The clean reads were used for mapping to the reference genome four times, and only the reads that mapped to the same position of the reference genome each time were retained in our next analysis. A binomial distribution test was performed to identify 5-methylcytosine for each cytosine site. Then the potential methylation sites were checked with the thresholds of coverage > 4 × depth and false discovery rate (FDR) < 0.05.

### Analysis of methylation level in transposons and differentially methylated regions (DMRs)

The average CpG methylation was detected in different gene regions, including upstream (a window size of 100 bp for 2 kb regions), the gene body, and downstream (a window size of 100 bp for 2 kb regions) of the coding regions. The transposons of 2nRR and 2nCC were predicted using RepeatMasker, and the average CpG methylation in the upstream and downstream transposon regions (2 kb) was calculated and plotted using R. The regions with different methylation were detected using MOABS [35]. The R packages DSS and bsseq were used to call DMRs between homoeologous gene pairs in the two triploids and between orthologous genes in 2nRR and 2nCC based on the threshold of e-value < 10^−5^. The functional enrichment of DMRs was performed by annotating them with the Gene Ontology and Kyoto Encyclopedia of Genes and Genomes databases with a threshold of *p*-value < 0.05.

### Association analyses of DNA methylation and homoeologous recombination

The promoter sequences (2 kb) of DMRs were identified for analyzing the differentially methylated genes (DMGs). The DMGs of orthologous gene pairs in the two inbred parents and homoeologous gene pairs in the hybrids were classified into the following two categories: (1) hyper-DMGs conforming to the thresholds of an absolute value of differences in the methylation ratio between 2nRR and 2nCC (|DMGs_A–B_|) > 0.6 and an absolute value of differences in the methylation ratios between the two homoeologs of the triploids (|DMGs_As–Bs_|) < 0.3, and 2) hypo-DMGs conforming to the thresholds of 0 <|DMGs_A–B_|< 0.6 in the inbred parents and |DMGs_As–Bs_|< 0.2 in the triploids.

### miRNA identification and differential expression

After DNase treatment, total RNA (~ 10 µg) of each sample was quality checked using a 2100 Bioanalyzer based on the values of the A260/A280 and A260/A230 ratios. Small RNAs were obtained by 6% polyacrylamide gel electrophoresis on Novex™ TBE gels (Invitrogen, Carlsbad, CA, USA) according to the manufacturer’s protocol. After adding adaptors, RT-PCR with adaptor-specific primers was used to generate a small RNA library from the two inbred parents (2nRR and 2nCC) and their two triploids (3nR_2_C and 3nRC_2_). The small RNA library was then sequenced by Illumina HiSeq 2500 (Illumina, San Diego, CA, USA) according to the manufacturer’s protocol.

Adaptor removal and quality control were performed by Cutadapt (v. 1.7.1), fastx_toolkit (v. 0.0.13) and NGSQCToolkit (v. 2.3.2). A similar strategy to methyl-seq was used for the mapping of miRNA reads. Subsequently, clean reads of 15–26 bp in 2nRR, 2nCC, and the two triploids were mapped to the combined genomes of 2nRR and 2nCC using Bowtie (v. 1.1.2) [36] and miRDeep2 (https://anaconda.org/bioconda/mirdeep2, v. 2.0.1.2) [37]. Then, the annotation of known miRNAs was performed by mapping to hairpin and mature miRNA sequences of zebrafish (http://www.mirbase.org/, Release 22.1). The annotated zebrafish miRNA files were downloaded from Ensembl database (http://dec2017.archive.ensembl.org/info/data/ftp/index.html). Then, the miRNA expression levels in the four fish were calculated using the script “quantifier.pl” in miRDeep2 software [37]. The number of mapped reads was obtained from the output results. The analyses of DE were performed using edgeR package [38] based on thresholds of |log_2_ fold change|> 1 and FDR < 0.01 for three biological replicates. The target genes of the miRNAs were obtained in two ways: (1) collection from miRTarBase database (http://mirtarbase.cuhk.edu.cn/php/index.php), and (2) prediction from miranda software [39].

### Copy number of miRNAs between red crucian carp and common carp

The miRNAs identified by the above analyses were used in our next analyses. Redundant sequences of 2nRR and 2nCC were deleted using CD-HIT (v. 4.8.1) software (https://github.com/weizhongli/cdhit/releases) with a threshold of sequence identity = 1.0. Then, the miRNA sequences were mapped to the combined genomes of 2nRR and 2nCC using the script “mapper.pl” of miRDeep2 software [36]. Among the genome locations of the miRNAs, potential homologous miRNAs were considered those with a distribution between the orthologous chromosomes of 2nRR and 2nCC. Lastly, the multiple locations of these potential homologous miRNAs were used to investigate the copy number of the homologous miRNAs.

### Expression of homoeologous genes

Muscle mRNA-seq data were downloaded from the Sequence Read Archive database in NCBI (accession numbers: SRS4475351–SRS4475354). These samples are the same as the ones in the miRNA and DNA methylation studies. Low-quality reads and adapters were trimmed off by Trimmomatic [40]. Then, the quality of the Illumina reads was checked with FastQC software (v. 0.11.7) [41]. A similar strategy to that of methyl-seq was used for mapping to investigate homoeologous gene expression. mRNA-seq reads were mapped to the combined genomes of *C. auratus* red var. [11] and *C. carpio* L. [24] using STAR software [42] with the parameters “–outSAMmapqUnique 255.” The unique mapped reads were collected using samtools [43] for the next expression analyses. Lastly, the read count was collected using HTSeq [44].

These genes were deleted in homoeolog expression analyses with the threshold of mapped read counts < 5 in each sample. The homoeolog expression of the two triploids was calculated based on the mapped read counts in the combined genome file, while expression levels of homologous genes in the two inbred parents were calculated based on the mapped results of the corresponding genome. The analyses of normalization and DE were performed using the edgeR package [38]. Differentially expressed genes (DEGs) were detected with the thresholds of |log_2_ FC|> 1 and FDR < 5% with three biological replicates.

### Obtaining mapped reads in homoeologous gene pairs using whole-genome resequencing

To investigate the homoeologous recombination (HRs) at the genomic level, whole-genome resequencing data were obtained from sequencing of HiSeq X Ten (paired end, 150 bp) in the NCBI Sequence Read Archive database [25]. Quality checking and adapter removal were performed with Trim Galore (v. 0.4.0) (http://www.bioinformatics.babraham.ac.uk/projects/trim_galore/) and Cutadapt (v. 1.2.1), respectively. Then, the high-quality reads were mapped to the combined genome of *C. auratus* and *C. carpio* using BWA (v. 0.7.17-r1188) with the default parameters. Then, the number of mapped reads in each gene was calculated using htseq-count (v. 0.12.4) with the threshold of “-m union–nonunique = none.” The HRs were assessed based on the ratio of uniquely mapped reads of homoeologs R vs. C [25].

## Supplementary Information

**Additional file 1: Fig. S1.** DNA methylation level of regions of TEs. A.TE distributions in each orthologous chromosomes of genomes R (2nRR) and C (2nCC). TE distributions were identified using 0.1 Mb sliding windows. B.The total methylation ratio (the combination of two homoeologs) in the two triploids and their inbred parents.C.The methylation ratio of homoeologs R vs.C in the two triploids and the total expressions in the two parents. **Fig. S2.** The cluster of DNA methylation of gene element among the two triploids and their inbred parents. DNA methylation in subgenomes R and C of the triploids. Black boxes indicate the DMRs, in which the similar trends of DNA methylation were in subgenomes/genomes R and C, respectively. Green box indicates the other DMRs in among these fishes. “Up in 2 k” represents 2 kb upstream of transcription start site. “Down in 2 k” represents 2 kb downstream of transcription termination site. “First exon” and “First intron” represent the first exon and intron in gene body, respectively. “Inner exon” represents all exons, except first and last exons. “Inner intron” represents all introns, except first intron. “Last exon” represents last exon in gene body. Each region was divided into twenty bins based on length. **Fig. S3.** GO annotation (Biological Process in level 3) of gene regulated by DNA methylation. **Fig. S4.** Correlation analyses between the values of differential expression (DE) and values of differential methylation (DM). A.Negative correlation between values of DE and DM (24 genes) in group of 3nR2C vs.2nCC.B.Negative correlation between values of DE and DM (5 genes) in group of 3nRC2vs.2nCC. C.Negative correlation between values of DE and DM (15 genes) in group of 3nR2C vs.2nRCC. D.Negative correlation between values of DE and DM (183 genes) in group of 3nRC2vs.2nRR. Red dot indicates the negative correlation between values of DE and DM. black dot indicates the positive correlation between values of DE and DM. **Fig. S5.** Differential expression (DE) analysis of miRNAs among the two triploids and their inbred parents. A.The number represents the number of up-regulated genes in the sample of the corresponding comparison. For example, the number “18” reflects that the 18 genes were higher expression in 2nRRthan in 3nRC2. B.The distribution of DE miRNAs in the five comparisons (3nR2Cvs.3nRC2, 3nR2Cvs. 2nRR, 3nRC2 vs.2nRR, 3nR2Cvs. 2nCC and 3nRC2 vs.2nCC). **Fig. S6.** GO annotation (Biological Process in level 3) in homoeologous recombinant genes (HRGs). **Fig. S7.** The distribution ofthe six DMGs in pathway of growth hormone synthesis, secretion and action (map04935). Red presents the hyper-DMGs. Green presents the HRGs in hypo-DMGs, while pink presents the other hypo-DMGs. *PRKCA*(K02677): classical protein kinase C alpha type; *ATF4*(K04374): cyclic AMP-dependent transcription factor ATF-4; *CACNA1F*(K04853): voltage-dependent calcium channel L type alpha-1F; *PLCB*(K05858): phosphatidylinositol phospholipase C, beta; *CREB1*(K05870): cyclic AMP-responsive element-binding protein 1; *STAT5A*(K11223): signal transducer and activator of transcription 5A. **Fig. S8.** The distribution of the nine DMGs in pathway of insulin secretion (map04911).Green presents the HRGs in hypo-DMGs, while pink presents the other hypo-DMGs. *ATP1B*(K01540): sodium/potassium-transporting ATPase subunit beta; *PRKCA*(K02677): classical protein kinase C alpha type; *ATF4*(K04374): cyclic AMP-dependent transcription factor ATF-4; *STX1A*(K04560): syntaxin 1A; *CACNA1F*(K04853): voltage-dependent calcium channel L type alpha-1F; *KCNN2*(K04943): potassium intermediate/small conductance calcium-activated channel subfamily N member 2; *PLCB*(K05858): phosphatidylinositol phospholipase C, beta; *CREB1*(K05870): cyclic AMP-responsive element-binding protein 1; *SNAP25*(K18211): synaptosomal-associated protein 25. **Table S1.** The mapping information of Methyl-seq data. **Table S2.** Summary of methylated and unmethylated cytosines. **Table S3.** The TE distribution of *C. auratus* red var. and *C. carpio haematopterus.* **Table S4.** The distribution of methylated and unmethylated cytosines (CpG) in the two triploids and their inbred parents. **Table S5.** The summary of miRNA-seq data. **Table S6.** The pathway distributions of hyper-DMGs and hypo-DMGs (more than 5 in each pathway).

## Data Availability

The raw reads of the DNA methylation data and small RNA sequencing data have been deposited in the National Genomics Data Center (NGDC) (https://bigd.big.ac.cn/gsub/) under the BioProject accession no. PRJCA003625. The mRNA sequencing and whole-genome resequencing data can be downloaded from NCBI Sequence Read Archive under the accession nos. SRR9983189, SRR9983190, SRR8735277–SRR8735279, SRR8712975–SRR8712976, SRR8712978–SRR8712991, SRR9203584, SRR9185089, and SRR9185090.

## Abbreviations

Mya: Million years ago
miRNAs: MicroRNAs
methyl-seq: DNA methylation sequencing
2nRR: Diploid *C. auratus* red var.
2nCC: Diploid *C. carpio* L.
4nR_2_C_2_: An allotetraploid of *C. auratus* red var. x *C. carpio* L.
3nR_2_C: Triploid fish obtained from hybridization between *C. auratus* red var. (♀) and an allotetraploid of *C. auratus* red var. x *C. carpio* L. (4nR_2_C_2_, ♂)
3nRC_2_: Triploid fish obtained from hybridization between *C. carpio* (♀) and 4nR_2_C_2_(♂)
WGBS: Whole-genome bisulfite sequencing
FDR: False discovery rate
DMRs: Differentially methylated regions
DMGs: Differentially methylated genes
DEGs: Differentially expressed genes
HRs: Homoeologous recombination
BL: Body length
BH: Body height
HBM: Height of back muscle
BW: Body weight
TEs: Transposable elements
OCP: Orthologous chromosome pair
TSS: Transcription start site
TTS: Transcription termination site
HRGs: Homoeologous recombinant genes
DE: Differential expression
DM: Differential methylation
NGDC: National Genomics Data Center
