# Supplementary material for: Heterosis of growth trait regulated by DNA methylation and miRNA in allotriploid fish
Source: Epigenetics Chromatin. 2022 May 21;15:19. doi: 10.1186/s13072-022-00455-6 (PMC9123727; doi:10.1186/s13072-022-00455-6)
Supplement: Supplementary file 1 — Additional file 1: Fig. S1. DNA methylation level of regions of TEs. A.TE distributions in each orthologous chromosomes of genomes R (2nRR) and C (2nCC). TE distributions were identified using 0.1 Mb sliding windows. B.The total methylation ratio (the combination of two homoeologs) in the two triploids and their inbred parents.C.The methylation ratio of homoeologs R vs.C in the two triploids and the total expressions in the two parents. Fig. S2. The cluster of DNA methylation of gene element among the two triploids and their inbred parents. DNA methylation in subgenomes R and C of the triploids. Black boxes indicate the DMRs, in which the similar trends of DNA methylation were in subgenomes/genomes R and C, respectively. Green box indicates the other DMRs in among these fishes. “Up in 2 k” represents 2 kb upstream of transcription start site. “Down in 2 k” represents 2 kb downstream of transcription termination site. “First exon” and “First intron” represent the first exon and intron in gene body, respectively. “Inner exon” represents all exons, except first and last exons. “Inner intron” represents all introns, except first intron. “Last exon” represents last exon in gene body. Each region was divided into twenty bins based on length. Fig. S3. GO annotation (Biological Process in level 3) of gene regulated by DNA methylation. Fig. S4. Correlation analyses between the values of differential expression (DE) and values of differential methylation (DM). A.Negative correlation between values of DE and DM (24 genes) in group of 3nR2C vs.2nCC.B.Negative correlation between values of DE and DM (5 genes) in group of 3nRC2vs.2nCC. C.Negative correlation between values of DE and DM (15 genes) in group of 3nR2C vs.2nRCC. D.Negative correlation between values of DE and DM (183 genes) in group of 3nRC2vs.2nRR. Red dot indicates the negative correlation between values of DE and DM. black dot indicates the positive correlation between values of DE and DM. Fig. S5. Differential exp [file 13072_2022_455_MOESM1_ESM.pdf]

Heterosis of growth trait regulated by DNA methylation and miRNA in allotriploid fish

Li Ren[1,2,#], Hong Zhang[1,2,#], Mengxue Luo[1,2], Xin Gao[1,2], Jialin Cui[1,2], Xueyin Zhang[1,2], Shaojun Liu[1,2,*]

[1]State Key Laboratory of Developmental Biology of Freshwater Fish, College of Life Sciences, Hunan Normal University, Changsha, Hunan, P.R. China.

[2]Guangdong Laboratory for Lingnan Modern Agriculture, South China Agricultural University, Guangzhou, 510642, Guangdong, PR China.

[*]Author for correspondence:

Professor Shaojun Liu: lsj@hunnu.edu.cn

State Key Laboratory of Developmental Biology of Freshwater Fish, Hunan Normal University, Changsha 410081, China

Tel/Fax: +86-731-88873074

[#]These authors contributed equally to this work.

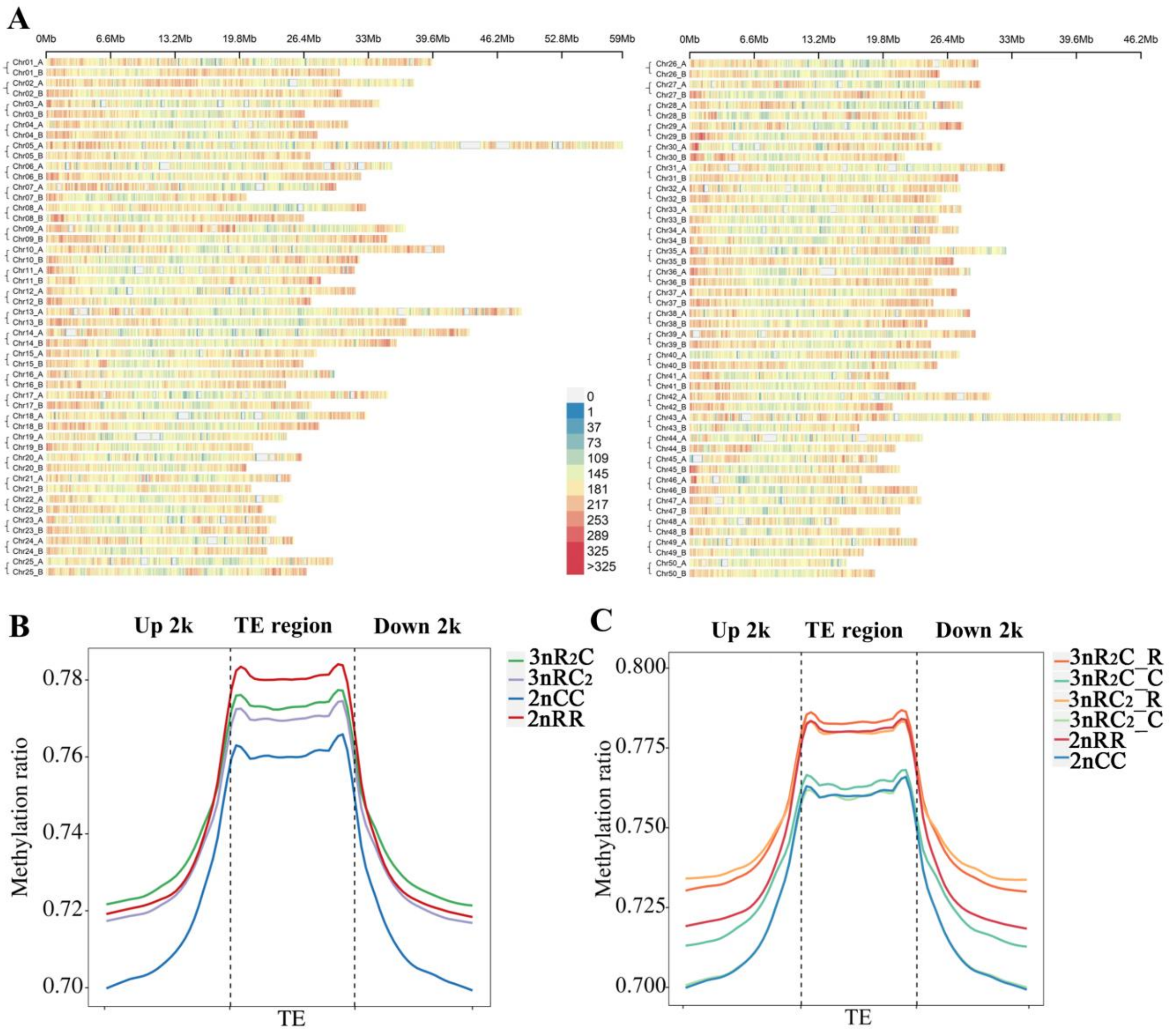

Fig. S1. DNA methylation level of regions of TEs. A. TE distributions in each orthologous chromosomes of genomes R (2nRR) and C (2nCC). TE distributions were identified using 0.1 Mb sliding windows. **B.** The total methylation ratio (the combination of two homoeologs) in the two triploids and their inbred parents. **C.** The methylation ratio of homoeologs R *vs.* C in the two triploids and the total expressions in the two parents.

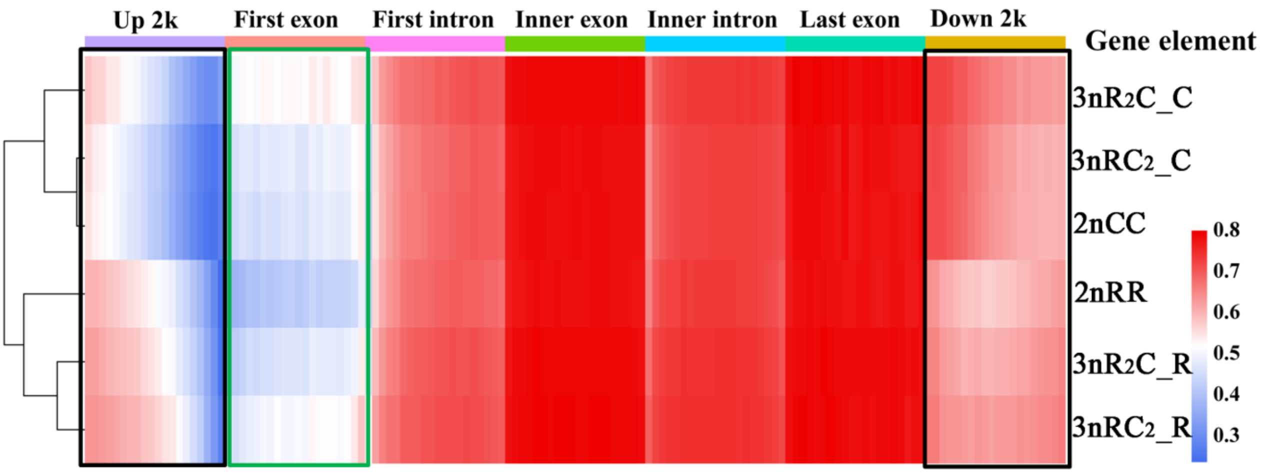

Fig. S2. The cluster of DNA methylation of gene element among the two triploids and their inbred parents. DNA methylation in subgenomes R and C of the triploids. Black boxes indicate the DMRs, in which the similar trends of DNA methylation were in subgenomes/genomes R and C, respectively. Green box indicates the other DMRs in among these fishes. "Up in 2k" represents 2 kb upstream of transcription start site. "Down in 2k" represents 2 kb downstream of transcription termination site. "First exon" and "First intron" represent the first exon and intron in gene body, respectively. "Inner exon" represents all exons, except first and last exons. "Inner intron" represents all introns, except first intron. "Last exon" represents last exon in gene body. Each region was divided into twenty bins based on length.

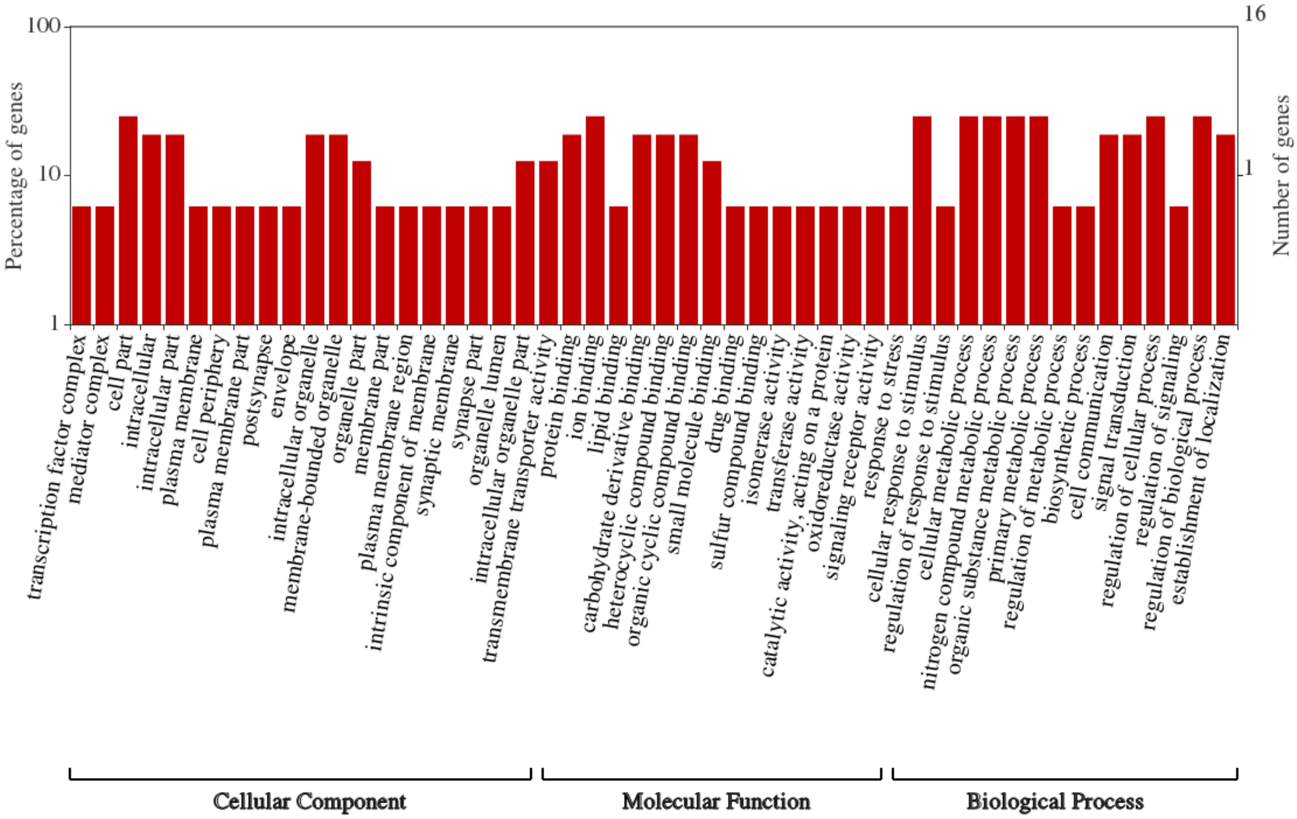

Fig. S3. GO annotation (Biological Process in level 3) of gene regulated by DNA methylation.

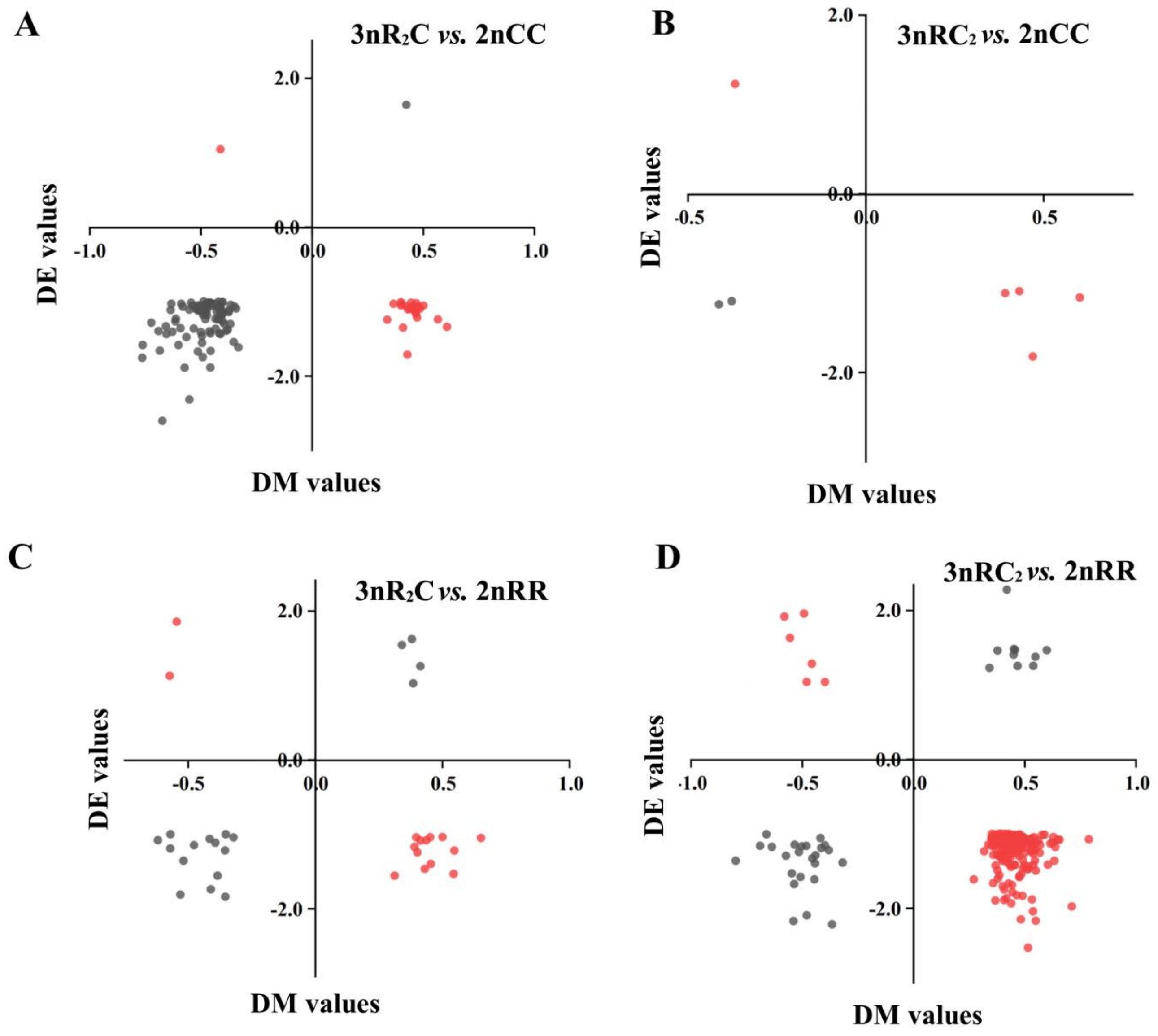

Fig. S4. Correlation analyses between the values of differential expression (DE) and values of differential methylation (DM). A. Negative correlation between values of DE and DM (24 genes) in group of $3nR_2C$ *vs.* 2nCC. **B.** Negative correlation between values of DE and DM (5 genes) in group of $3nRC_2$ *vs.* 2nCC. **C.** Negative correlation between values of DE and DM (15 genes) in group of $3nR_2C$ *vs.* 2nRCC. **D.** Negative correlation between values of DE and DM (183 genes) in group of $3nRC_2$ *vs.* 2nRR. Red dot indicates the negative correlation between values of DE and DM. black dot indicates the positive correlation between values of DE and DM.

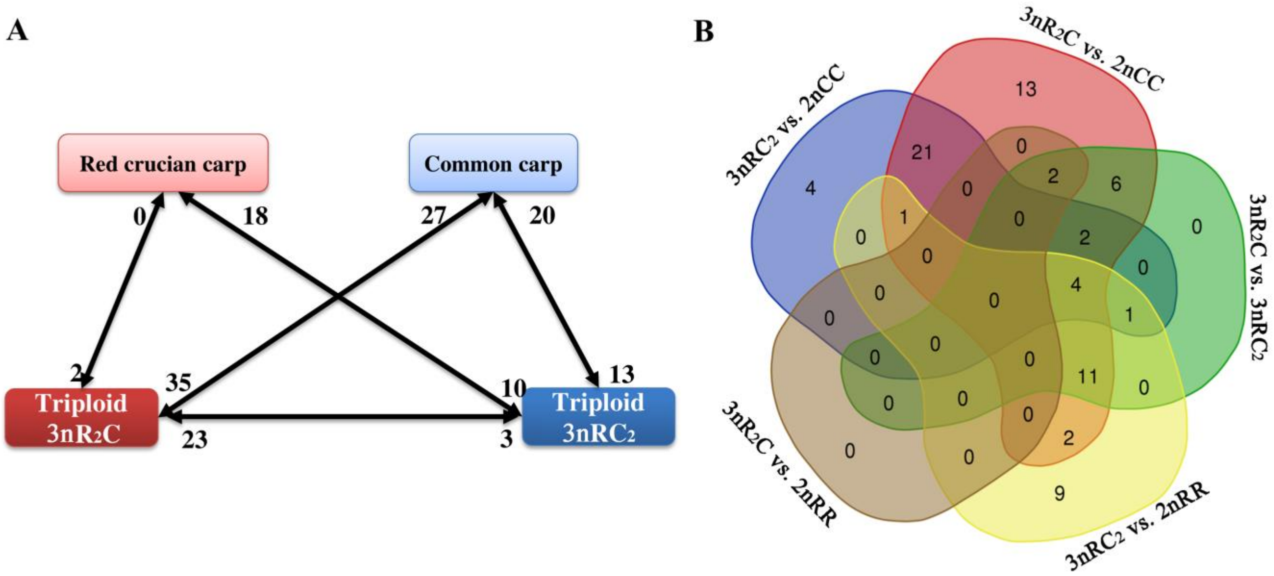

Fig. S5. Differential expression (DE) analysis of miRNAs among the two triploids and their inbred parents. A. The number represents the number of up-regulated genes in the sample of the corresponding comparison. For example, the number "18" reflects that the 18 genes were higher expression in 2nRR than in 3nRC$_2$. **B.** The distribution of DE miRNAs in the five comparisons (3nR$_2$C *vs.* 3nRC$_2$, 3nR$_2$C *vs.* 2nRR, 3nRC$_2$ *vs.*2nRR, 3nR$_2$C *vs.* 2nCC and 3nRC$_2$ *vs.* 2nCC).

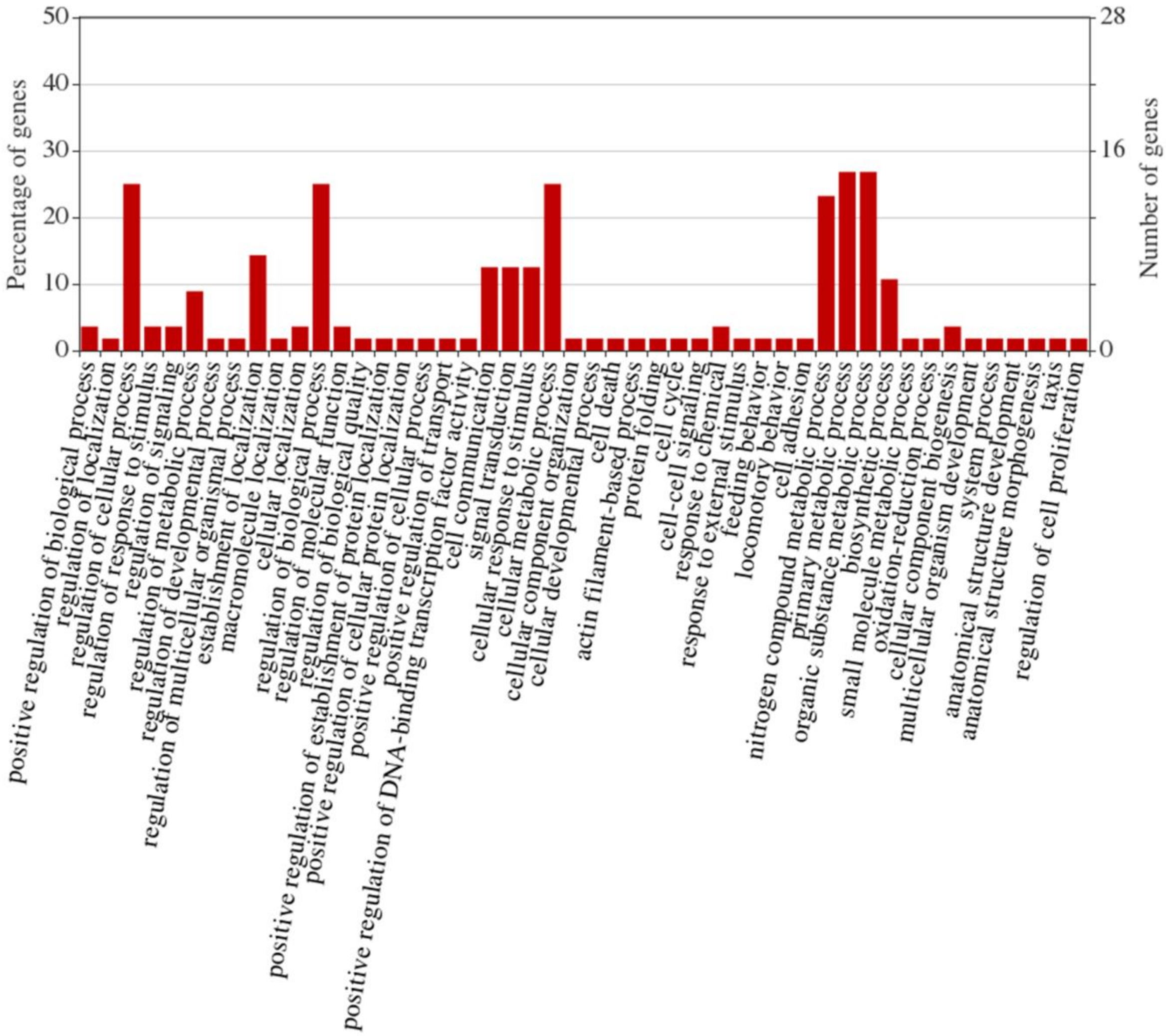

Fig. S6. GO annotation (Biological Process in level 3) in homoeologous recombinant genes (HRGs).

Growth hormone synthesis, secretion and action (map04935)

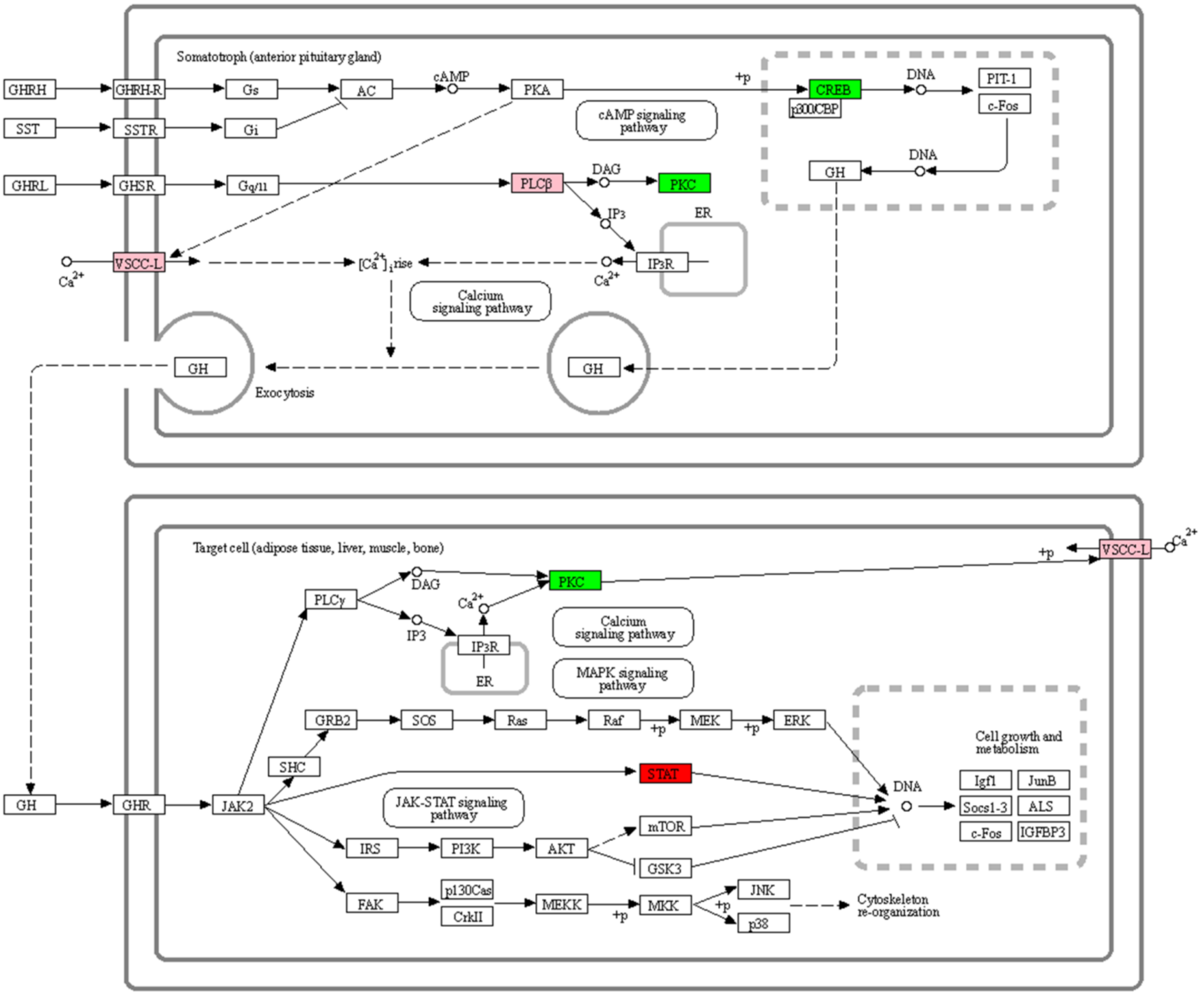

Fig. S7. The distribution of the six DMGs in pathway of growth hormone synthesis, secretion and action (map04935). Red presents the hyper-DMGs. Green presents the HRGs in hypo-DMGs, while pink presents the other hypo-DMGs. *PRKCA* (K02677): classical protein kinase C alpha type; *ATF4* (K04374): cyclic AMP-dependent transcription factor ATF-4; *CACNA1F* (K04853): voltage-dependent calcium channel L type alpha-1F; *PLCB* (K05858): phosphatidylinositol phospholipase C, beta; *CREB1* (K05870): cyclic AMP-responsive element-binding protein 1; *STAT5A* (K11223): signal transducer and activator of transcription 5A.

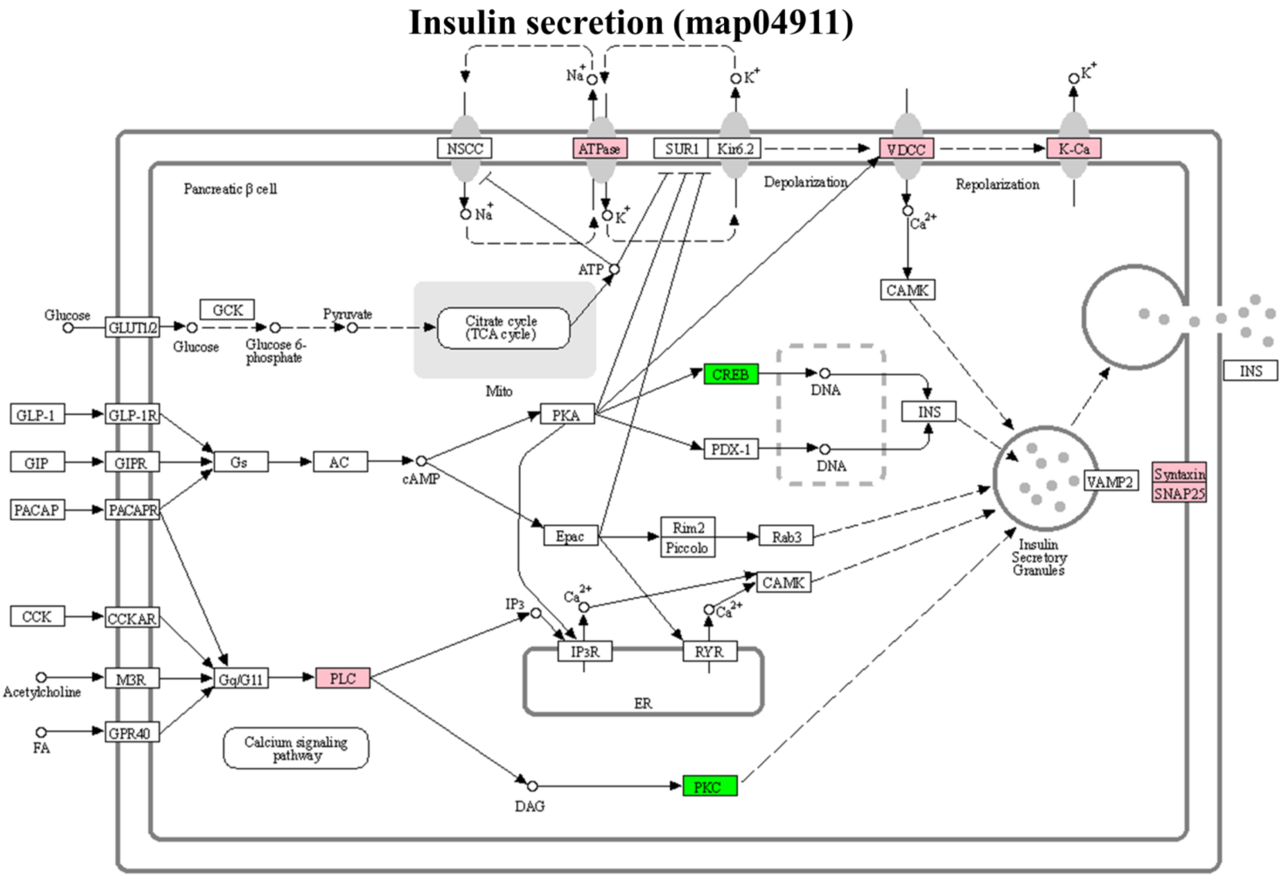

Fig. S8. The distribution of the nine DMGs in pathway of insulin secretion (map04911). Green presents the HRGs in hypo-DMGs, while pink presents the other hypo-DMGs. *ATP1B* (K01540): sodium/potassium-transporting ATPase subunit beta; *PRKCA* (K02677): classical protein kinase C alpha type; *ATF4* (K04374): cyclic AMP-dependent transcription factor ATF-4; *STX1A* (K04560): syntaxin 1A; *CACNA1F* (K04853): voltage-dependent calcium channel L type alpha-1F; *KCNN2* (K04943): potassium intermediate/small conductance calcium-activated channel subfamily N member 2; *PLCB* (K05858): phosphatidylinositol phospholipase C, beta; *CREB1* (K05870): cyclic AMP-responsive element-binding protein 1; *SNAP25* (K18211): synaptosomal-associated protein 25.

Table S1. The mapping information of Methyl-seq data.

	No. of total reads	Total data (Gb)	No. unique mapped reads	Unique mapped data (Gb)	Unique mapped ratio
$3nR_2C$-1	674,844,366	101.23	331,673,338	49.75	49.10%
$3nR_2C$-2	571,295,500	85.69	242,647,864	36.40	42.50%
$3nR_2C$-3	627,271,854	94.09	346,359,296	51.95	55.20%
$3nRC_2$-1	602,815,714	90.42	295,684,799	44.35	48.98%
$3nRC_2$-2	589,584,956	88.44	269,073,242	40.36	45.60%
$3nRC_2$-3	615,028,722	92.25	320,249,314	48.04	52.10%
2nCC-1	632,903,686	94.94	290,492,450	43.57	45.90%
2nCC-2	621,020,180	93.15	297,179,314	44.58	47.90%
2nCC-3	617,609,614	92.64	334,919,330	50.24	54.20%
2nRR-1	609,661,984	91.45	321,190,676	48.18	52.70%
2nRR-2	607,502,680	91.13	325,157,448	48.78	53.50%
2nRR-3	619,864,446	92.98	359,255,464	53.89	58.00%
Total	7,389,403,702	1,108	3,733,882,535	/	/

Table S2. Summary of methylated and unmethylated cytosines.

		No. of methylation cytosines	No. of unmethylation cytosines	Ratio of methylation cytosines	No. of total cytosines
$3nR_2C$-1	CpG	427,870,647	164,151,618	72.30%	7,460,844,480
	CHG	4,797,278	1,569,618,669	0.30%	
	CHH	15,359,633	5,279,046,635	0.30%	
$3nR_2C$-2	CpG	293,881,554	116,926,822	71.50%	5,351,787,576
	CHG	3,390,077	1,120,416,639	0.30%	
	CHH	10,982,722	3,806,189,762	0.30%	
$3nR_2C$-3	CpG	481,171,975	196,615,172	71.00%	8,180,415,790
	CHG	4,982,603	1,765,191,228	0.30%	
	CHH	15,929,721	5,716,525,091	0.30%	
$3nRC_2$-1	CpG	369,719,091	156,202,089	70.38%	6,748,903,622
	CHG	4,264,746	1,446,309,075	0.30%	

	CHH	13,786,166	4,758,622,455	0.30%	
	CpG	297,329,777	121,769,170	70.90%	
3nRC$_2$-2	CHG	3,781,283	1,203,389,288	0.30%	5,790,280,678
	CHH	12,469,366	4,151,541,794	0.30%	
	CpG	436,539,996	187,986,321	69.90%	
3nRC$_2$-3	CHG	4,711,020	1,670,542,725	0.30%	7,633,786,340
	CHH	15,001,674	5,319,004,604	0.30%	
	CpG	340,982,176	134,100,392	71.80%	
2nCC-1	CHG	4,574,448	1,324,356,940	0.30%	6,362,028,956
	CHH	14,627,293	4,543,387,707	0.30%	
	CpG	322,750,223	137,718,500	70.10%	
2nCC-2	CHG	4,523,446	1,369,908,417	0.30%	6,523,670,409
	CHH	14,985,210	4,673,784,613	0.30%	
	CpG	418,871,287	189,590,083	68.80%	
2nCC-3	CHG	5,563,161	1,705,986,331	0.30%	7,860,661,214
	CHH	18,015,670	5,522,634,682	0.30%	
	CpG	403,184,485	168,876,116	70.50%	
2nRR-1	CHG	4,097,629	1,489,054,923	0.30%	7,124,141,309
	CHH	12,882,068	5,046,046,088	0.30%	
	CpG	419,828,068	151,084,118	73.50%	
2nRR-2	CHG	4,228,121	1,489,264,552	0.30%	7,140,981,310
	CHH	13,604,324	5,062,972,127	0.30%	
	CpG	530,480,797	207,041,775	71.90%	
2nRR-3	CHG	4,518,511	1,833,489,790	0.20%	8,514,337,726
	CHH	14,121,811	5,924,685,042	0.20%	

Table S3. The TE distribution of *C. auratus* red var. and *C. carpio haematopterus*

	C. auratus red var.				*C. carpio haematopterus*				Difference rate (%)
	Chromosome ID	Number	Length (bp)	Rate (%)	Chromosome ID	Number	Length (bp)	Rate (%)	

OCP 1	chr6	67,237	16,266,412	41.16	GWHAATB00000001	55,795	11,151,639	37.14	4.02
OCP 2	chr7	65,876	16,982,485	45.12	GWHAATB00000002	54,948	10,633,150	35.08	10.04
OCP 3	chr12	55,744	13,114,253	38.40	GWHAATB00000003	46,946	9,478,231	35.82	2.58
OCP 4	chr19	50,840	12,390,321	40.10	GWHAATB00000004	49,030	9,928,847	35.76	4.34
OCP 5	chr1	95,183	26,858,658	45.51	GWHAATB00000005	47,827	9,575,860	35.45	10.06
OCP 6	chr10	53,722	13,087,474	36.97	GWHAATB00000006	58,804	12,668,966	39.33	2.36
OCP 7	chr21	48,137	11,949,790	40.26	GWHAATB00000007	35,264	7,148,423	34.84	5.42
OCP 8	chr14	54,151	15,745,130	48.11	GWHAATB00000008	46,984	10,913,065	41.37	6.74
OCP 9	chr9	61,702	13,706,973	37.27	GWHAATB00000009	63,170	12,840,423	36.64	0.63
OCP 10	chr5	68,107	16,444,752	40.35	GWHAATB00000010	56,650	10,872,090	33.85	6.50
OCP 11	chr17	48,945	10,274,573	32.55	GWHAATB00000011	48,913	9,349,312	33.29	0.74
OCP 12	chr18	50,984	11,603,558	36.63	GWHAATB00000012	46,358	8,587,854	31.64	4.99
OCP 13	chr2	77,325	19,921,993	40.94	GWHAATB00000013	63,920	12,964,551	35.03	5.91
OCP 14	chr3	70,713	16,665,321	38.42	GWHAATB00000014	62,474	12,813,038	35.69	2.73
OCP 15	chr32	46,737	10,722,967	38.73	GWHAATB00000015	47,189	9,441,609	35.88	2.85
OCP 16	chr25	50,705	11,513,284	39.05	GWHAATB00000016	44,275	8,475,037	34.55	4.50
OCP 17	chr11	56,263	13,456,237	38.42	GWHAATB00000017	47,285	9,219,078	33.96	4.46
OCP 18	chr13	51,272	12,279,809	37.47	GWHAATB00000018	49,720	9,459,851	33.84	3.63
OCP 19	chr45	36,284	8,112,030	32.95	GWHAATB00000019	36,271	7,064,681	33.38	0.43
OCP 20	chr39	40,135	9,017,095	34.46	GWHAATB00000020	35,316	6,738,241	32.91	1.55
OCP 21	chr44	41,865	9,128,923	36.44	GWHAATB00000021	36,538	7,054,287	33.60	2.84
OCP 22	chr48	40,448	9,137,884	37.80	GWHAATB00000022	39,373	7,622,676	34.25	3.55
OCP 23	chr33	37,990	8,639,792	36.68	GWHAATB00000023	40,679	7,659,971	33.57	3.11
OCP 24	chr42	41,715	9,451,459	37.24	GWHAATB00000024	39,423	7,455,570	32.89	4.35
OCP 25	chr26	46,060	10,912,301	37.18	GWHAATB00000025	46,289	9,071,880	34.05	3.13
OCP 26	chr23	48,605	10,889,596	36.86	GWHAATB00000026	43,883	8,112,018	31.68	5.18
OCP 27	chr22	48,404	11,284,555	37.84	GWHAATB00000027	41,522	8,072,322	33.46	4.38

OCP 28	chr31	45,136	10,405,156	37.22	GWHAATB00000028	41,411	8,088,775	33.31	3.91
OCP 29	chr29	45,485	11,126,907	39.70	GWHAATB00000029	43,726	8,966,548	37.04	2.66
OCP 30	chr24	42,588	10,834,857	41.89	GWHAATB00000030	39,611	7,713,047	35.03	6.86
OCP 31	chr16	49,993	12,096,128	37.38	GWHAATB00000031	48,041	9,670,620	35.17	2.21
OCP 32	chr15	46,260	11,146,349	40.21	GWHAATB00000032	46,144	8,927,252	34.60	5.61
OCP 33	chr28	44,237	10,123,604	36.26	GWHAATB00000033	42,975	8,166,409	32.03	4.23
OCP 34	chr35	44,575	9,809,733	35.62	GWHAATB00000034	42,032	7,912,175	32.18	3.44
OCP 35	chr47	53,912	13,557,986	41.84	GWHAATB00000035	49,664	10,095,810	37.36	4.48
OCP 36	chr8	48,836	11,427,991	39.76	GWHAATB00000036	44,755	9,124,122	36.68	3.08
OCP 37	chr36	48,299	11,398,539	41.68	GWHAATB00000037	44,750	8,715,255	34.96	6.72
OCP 38	chr30	49,206	11,173,993	38.93	GWHAATB00000038	43,428	8,610,054	35.39	3.54
OCP 39	chr27	47,893	10,398,801	35.54	GWHAATB00000039	43,754	8,360,090	33.83	1.71
OCP 40	chr34	45,639	10,441,130	37.78	GWHAATB00000040	44,880	8,741,402	34.47	3.31
OCP 41	chr50	33,518	7,404,217	36.12	GWHAATB00000041	41,149	8,378,136	35.97	0.15
OCP 42	chr20	49,885	12,241,256	39.67	GWHAATB00000042	36,730	7,052,485	33.91	5.76
OCP 43	chr4	73,276	22,491,040	50.98	GWHAATB00000043	28,769	5,936,070	34.23	16.75
OCP 44	chr40	34,608	8,724,363	36.56	GWHAATB00000044	37,161	7,977,281	37.79	1.23
OCP 45	chr41	30,375	7,174,192	37.33	GWHAATB00000045	37,753	7,130,995	33.09	4.24
OCP 46	chr37	29,447	6,883,316	39.05	GWHAATB00000046	41,397	8,006,228	34.36	4.69
OCP 47	chr43	38,649	8,784,613	37.01	GWHAATB00000047	37,964	7,619,890	35.37	1.64
OCP 48	chr38	23,829	6,455,638	42.20	GWHAATB00000048	38,402	7,558,126	34.91	7.29
OCP 49	chr46	38,925	9,965,567	42.75	GWHAATB00000049	30,153	6,014,857	33.78	8.97
OCP 50	chr49	24,644	6,640,227	41.22	GWHAATB00000050	33,064	6,415,357	33.81	7.41
Total		2,444,364	590,263,228			2,222,559	439,553,654		
Average		48,887	11,805,265	38.99		44,451	8,791,073	34.76	4.23

Note: OCP, Orthologous chromosome pair.

Table S4. The distribution of methylated and unmethylated cytosines (CpG) in the two triploids and their inbred parents.

		No. of methylation cytosines	No. of unmethylation cytosines	Methylation rate	Different rate[a]
	subgenome R	301,956,100	111,250,411	73.08%	
$3nR_2C$-1	subgenome C	125,914,547	52,901,207	70.42%	2.66%
	Total	427,870,647	164,151,618	72.30%	
	subgenome R	202,679,415	77,503,575	72.34%	
$3nR_2C$-2	subgenome C	91,202,139	39,423,247	69.82%	2.52%
	Total	293,881,554	116,926,822	71.50%	
	subgenome R	337,153,506	132,549,092	71.78%	
$3nR_2C$-3	subgenome C	144,018,469	64,066,080	69.21%	2.57%
	Total	481,171,975	196,615,172	71.00%	
	subgenome R	139,014,378	54,514,529	71.83%	
$3nRC_2$-1	subgenome C	230,704,713	101,687,560	69.41%	2.42%
	Total	369,719,091	156,202,089	70.38%	
	subgenome R	112,141,515	42,541,409	72.50%	
$3nRC_2$-2	subgenome C	185,188,262	79,227,761	70.04%	2.46%
	Total	297,329,777	121,769,170	70.90%	
	subgenome R	164,127,132	65,497,446	71.48%	
$3nRC_2$-3	subgenome C	272,412,864	122,488,875	68.98%	2.50%
	Total	436,539,996	187,986,321	69.90%	
2nRR-1	genome R	403,184,485	168,876,116	70.50%	
2nRR-2	genome R	419,828,068	151,084,118	73.50%	
2nRR-3	genome R	530,480,797	207,041,775	71.90%	2.52%
2nCC-1	genome C	340,982,176	134,100,392	71.80%	
2nCC-2	genome C	322,750,223	137,718,500	70.10%	
2nCC-3	genome C	418,871,287	189,590,083	68.80%	

Note: [a] represents different rate between subgenome R and C in the triploids, or different rate between genome R and C in the inbred parents.

Table S5. The summary of miRNA-seq data.

	Number of clean data	Total bases of clean data (MB)	Number of mapped reads (≥ 1)	Mapped percent
$3nR_2C$-1	9,961,199	221.99	9,275,869	93.12%
$3nR_2C$-2	11,392,855	254.65	10,847,137	95.21%
$3nR_2C$-3	10,764,944	238.68	9,966,185	92.58%
$3nRC_2$-1	10,374,423	233.51	9,487,410	91.45%
$3nRC_2$-2	10,828,495	248.01	9,953,553	91.92%
$3nRC_2$-3	11,091,146	244.46	10,140,635	91.43%
2nCC-1	10,648,373	251.34	10,222,438	96.00%
2nCC-2	9,721,015	237.19	9,160,112	94.23%
2nCC-3	11,235,909	249.03	10,666,248	94.93%
2nRR-1	10,327,240	230.15	9,985,408	96.69%
2nRR-2	10,296,407	227.43	9,732,164	94.52%
2nRR-3	12,443,982	277.49	11,662,500	93.72%
Total	129,085,988	2,914.93	121,099,659	/

Table S6. The pathway distributions of hyper-DMGs and hypo-DMGs (more than 5 in each pathway).

Pathway ID	Pathway name	Gene number
map01100	Metabolic pathways	47
map05200	Pathways in cancer	27
map04080	Neuroactive ligand-receptor interaction	20
map04144	Endocytosis	17
map04151	PI3K-Akt signaling pathway	17
map01110	Biosynthesis of secondary metabolites	16
map04010	MAPK signaling pathway	16
map04810	Regulation of actin cytoskeleton	16
map05417	Lipid and atherosclerosis	15
map04020	Calcium signaling pathway	14
map05022	Pathways of neurodegeneration - multiple diseases	14
map05202	Transcriptional misregulation in cancer	14
map05205	Proteoglycans in cancer	14
map04261	Adrenergic signaling in cardiomyocytes	13
map04152	AMPK signaling pathway	12
map04360	Axon guidance	12
map04728	Dopaminergic synapse	12
map04921	Oxytocin signaling pathway	12
map05163	Human cytomegalovirus infection	12
map05165	Human papillomavirus infection	12
map05171	Coronavirus disease - COVID-19	12
map05203	Viral carcinogenesis	12
map04024	cAMP signaling pathway	11
map04071	Sphingolipid signaling pathway	11
map04510	Focal adhesion	11

map05166	Human T-cell leukemia virus 1 infection	11
map05206	MicroRNAs in cancer	11
map04014	Ras signaling pathway	10
map04022	cGMP-PKG signaling pathway	10
map04725	Cholinergic synapse	10
map05014	Amyotrophic lateral sclerosis	10
map05161	Hepatitis B	10
map05167	Kaposi sarcoma-associated herpesvirus infection	10
map04015	Rap1 signaling pathway	9
map04072	Phospholipase D signaling pathway	9
map04270	Vascular smooth muscle contraction	9
map04724	Glutamatergic synapse	9
map04726	Serotonergic synapse	9
map04911	Insulin secretion	9
map04912	GnRH signaling pathway	9
map05010	Alzheimer disease	9
map03010	Ribosome	8
map04060	Cytokine-cytokine receptor interaction	8
map04140	Autophagy - animal	8
map04310	Wnt signaling pathway	8
map04514	Cell adhesion molecules	8
map04659	Th17 cell differentiation	8
map04713	Circadian entrainment	8
map04723	Retrograde endocannabinoid signaling	8
map04915	Estrogen signaling pathway	8
map04925	Aldosterone synthesis and secretion	8
map04926	Relaxin signaling pathway	8
map04928	Parathyroid hormone synthesis, secretion and action	8
map04933	AGE-RAGE signaling pathway in diabetic complications	8
map04976	Bile secretion	8
map05016	Huntington disease	8
map05130	Pathogenic Escherichia coli infection	8
map05131	Shigellosis	8
map05132	Salmonella infection	8
map05168	Herpes simplex virus 1 infection	8
map05170	Human immunodeficiency virus 1 infection	8
map00230	Purine metabolism	7
map04068	FoxO signaling pathway	7
map04070	Phosphatidylinositol signaling system	7
map04141	Protein processing in endoplasmic reticulum	7
map04520	Adherens junction	7
map04621	NOD-like receptor signaling pathway	7
map04630	JAK-STAT signaling pathway	7
map04666	Fc gamma R-mediated phagocytosis	7

map04727	GABAergic synapse	7
map04919	Thyroid hormone signaling pathway	7
map04924	Renin secretion	7
map04934	Cushing syndrome	7
map05020	Prion disease	7
map05031	Amphetamine addiction	7
map05034	Alcoholism	7
map05135	Yersinia infection	7
map05215	Prostate cancer	7
map04064	NF-kappa B signaling pathway	6
map04110	Cell cycle	6
map04217	Necroptosis	6
map04218	Cellular senescence	6
map04371	Apelin signaling pathway	6
map04530	Tight junction	6
map04550	Signaling pathways regulating pluripotency of stem cells	6
map04658	Th1 and Th2 cell differentiation	6
map04660	T cell receptor signaling pathway	6
map04721	Synaptic vesicle cycle	6
map04722	Neurotrophin signaling pathway	6
map04922	Glucagon signaling pathway	6
map04927	Cortisol synthesis and secretion	6
map04931	Insulin resistance	6
map04935	Growth hormone synthesis, secretion and action	6
map05032	Morphine addiction	6
map05162	Measles	6
map05219	Bladder cancer	6
map05231	Choline metabolism in cancer	6
